# An Updated and Revised Dyadic Model of Partner Violence

**DOI:** 10.3390/bs16091468

**Published:** 2026-08-24

**Authors:** Bahare Mazinani, Brian P. O’Connor

**Affiliations:** Department of Psychology, The University of British Columbia, Kelowna, BC V1V 1V7, Canada; bmzinani@student.ubc.ca

**Keywords:** intimate partner violence, dyadic model of partner violence, perpetration, victimization, physical IPV, risk markers

## Abstract

Research on intimate partner violence (IPV) has traditionally examined perpetration and victimization separately, limiting understanding of the relational processes that sustain violence. The present selective review builds on the dyadic model of partner violence by synthesizing meta-analytic evidence concerning risk markers for physical IPV. Eight peer-reviewed meta-analyses published between 2000 and 2024 formed the core synthesis. Studies were included if they reported quantitative effect sizes for variables associated with physical IPV perpetration or victimization. Extracted results were organized into three hierarchical levels: (a) background and dispositional risk markers, (b) relationship context, and (c) situational context. Across the reviewed meta-analyses, the most consistent risk markers of IPV clustered within the relationship-context level, particularly prior partner aggression, emotional abuse, and relationship dissatisfaction. Communication skills and empathy were inversely associated with IPV risk. Few studies examined same-sex couples, IPV victimization among men, or culturally diverse samples, highlighting major gaps in current evidence. This selective review provides an updated synthesis and conceptual organization of IPV risk markers and emphasizes the importance of dyadic frameworks for guiding future theory, prevention, and policy.

## 1. Introduction

Intimate partner violence (IPV) is defined by the Centers for Disease Control and Prevention (CDC) as “physical violence, sexual violence, stalking, and psychological aggression (including coercive acts) by a current or former intimate partner (e.g., current or former spouses, boyfriends, girlfriends, and dating or sexual partners)” (Breiding et al., 2015). Similarly, the World Health Organization (WHO) defines IPV as encompassing physical and sexual violence, emotional and psychological abuse, and controlling behaviors between partners (World Health Organization, 2012). Although the definitions substantially overlap, they differ in scope and emphasis. The CDC uses a surveillance-oriented, gender-neutral taxonomy that explicitly identifies physical violence, sexual violence, stalking, and psychological aggression, including coercive tactics, by a current or former intimate partner (Breiding et al., 2015). The WHO defines IPV as behavior by an intimate partner or ex-partner that causes physical, sexual, or psychological harm, including physical aggression, sexual coercion, psychological abuse, and controlling behaviors (World Health Organization, 2012). The WHO information sheet is framed primarily around violence against women, whereas the CDC surveillance definition is gender-neutral. One classificatory difference is that the CDC lists stalking as a distinct IPV category, whereas the WHO definition does not enumerate stalking separately; WHO explicitly includes controlling behaviors, which the CDC addresses within psychological aggression and coercive tactics. These differences are classificatory rather than contradictory: both definitions recognize physical, sexual, and psychological abuse and coercive control, and both encompass current and former partners. The present review focuses on physical IPV within this shared definitional core while recognizing that different forms of IPV frequently co-occur. IPV is multifaceted and often involves overlapping sexual, psychological, and physical forms. Physical IPV includes actions such as slapping, pushing, hitting, kicking, suffocation, or using a weapon against an intimate partner (Devries et al., 2013). Physical IPV is particularly serious because it can cause severe injury or death. Moreover, physical IPV may be more observable to third parties than sexual or psychological abuse. In this review, the term risk marker is used as a descriptive umbrella label for a characteristic or condition statistically associated with IPV when temporal precedence and causality have not been established. We use correlate for an explicitly non-directional association. Consistent with the distinctions proposed by Kraemer et al. (1997), risk factor is reserved for a variable shown to precede the outcome, whereas predictor is used only when a source reports prospective statistical prediction. Neither term, by itself, implies causation. The terms causal risk factor and mechanism are used only when the evidence and study design support a causal inference. Because temporal ordering is unclear for many of the associations summarized in the source meta-analyses, risk marker and association are the default terms throughout this review. A substantial body of literature has identified risk markers associated with physical IPV, aiding in the assessment, prevention, and intervention of such violence. However, partner violence is fundamentally an interpersonal phenomenon, shaped by the individual characteristics of each partner and the relational dynamics within couples (Conroy, 2014; Ghuman et al., 2006; Rodriguez et al., 2017). Although these dynamics operate at the couple level, much of the existing research has primarily focused on individuals as the unit of analysis (Behrman & Frye, 2021). Consequently, a couple-based approach offers a more comprehensive understanding of risk markers associated with IPV. Such an approach acknowledges that each partner brings distinct lived experiences and perspectives to the relationship, which interact dynamically and evolve over the course of the relationship (Behrman & Frye, 2021). This review builds on the dyadic model of partner violence proposed by Bartholomew and Cobb (2011) and updates it in two key respects. First, it organizes risk markers associated with both IPV perpetration and victimization. Second, it incorporates consideration of gender differences. Focusing on physical IPV in heterosexual relationships, the review emphasizes meta-analytic findings.

## 2. Theoretical Framework

### 2.1. The Dyadic Model of Partner Violence

Bartholomew and Cobb (2011) conceptualized relationship violence as an interpersonal process between two intimate partners. These authors recognized that IPV is fundamentally a relational phenomenon that emerges from the complex interplay of both partners’ characteristics and behaviors within a specific situational context. Their model is a multilevel framework that explains IPV by integrating various individual, relational, and situational factors. The model emphasizes the interdependence of partners’ characteristics and behaviors and the dynamic interplay of risk markers across its levels in relation to the occurrence or persistence of IPV episodes (Bartholomew & Cobb, 2011).

The first level focuses on individual background and dispositional characteristics, recognizing how personal traits and lived experiences shape each partner’s propensity for violence. The second level examines the relationship context, focusing on how partners’ predispositions interact to shape their relationship dynamics. This level emphasizes the quality of the relationship arising from the interaction between the two partners and their enduring characteristics. The third level addresses the situational context, examining the proximal circumstances surrounding episodes of IPV.

Together, the interplay of these three levels culminates in a distinct pattern of partner violence, which constitutes the fourth level. This level considers the severity of violence, whether it is mutual or one-sided, how partners respond to it, and the resulting consequences (Bartholomew & Cobb, 2011). According to Bartholomew and Cobb (2011), couple members reciprocally influence each other at each level of this model. As interpersonal interactions progress through the model, subsequent levels can feed back into earlier ones. Risk factors at earlier levels are generally expected to influence IPV through mediation by later levels.

**Figure 1:**
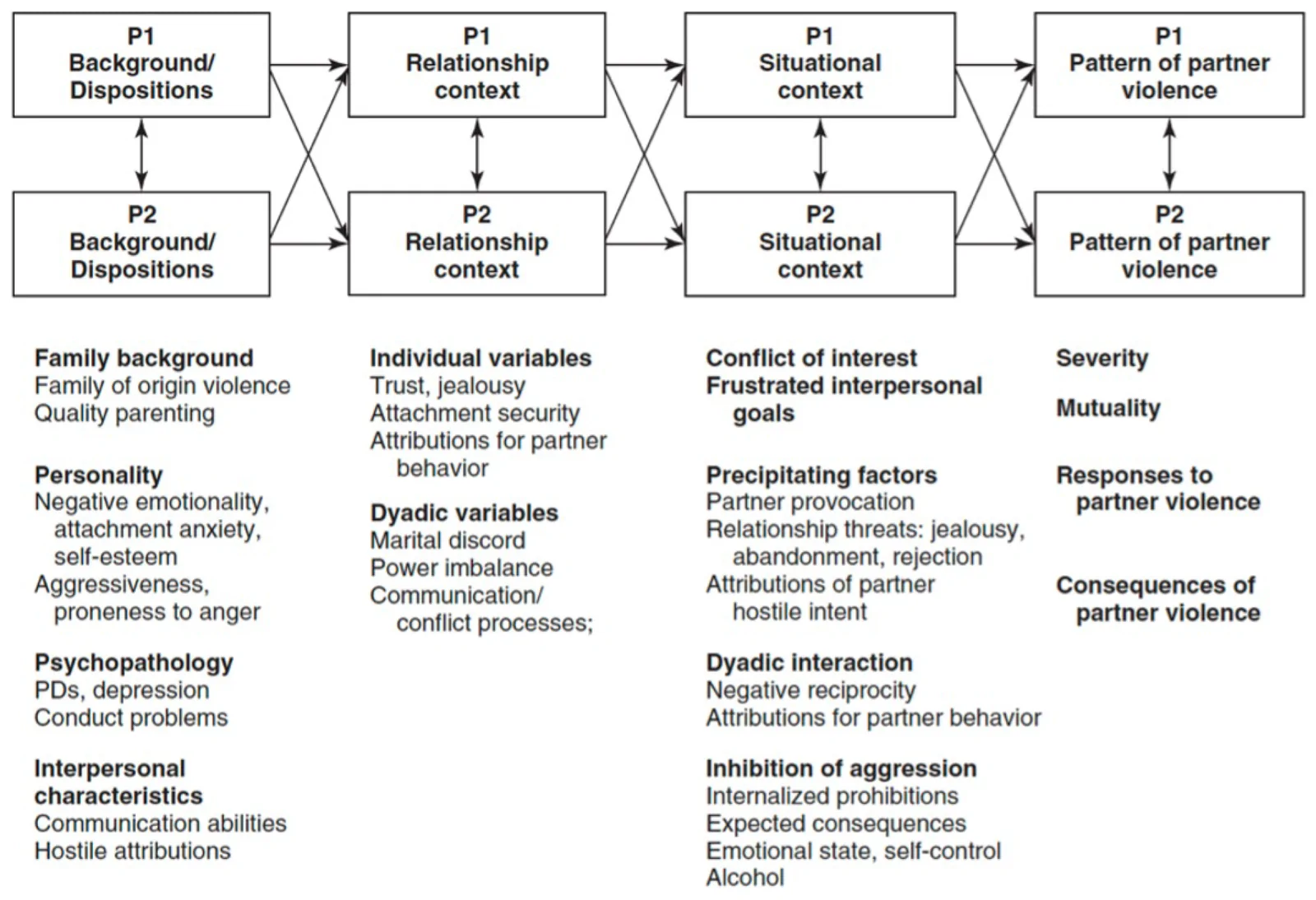
A Dyadic Model of Partner Violence. Reprinted from Conceptualizing Relationship Violence as a Dyadic Process by Bartholomew and Cobb (2011), in Handbook of Interpersonal Psychology: Theory, Research, Assessment, and Therapeutic Interventions. Copyright 2011 by John Wiley & Sons, Inc. Reprinted with permission.

In sum, the occurrence and persistence of violence within a dyad are not solely determined by each partner’s individual dispositions or background. Rather, they reflect a complex interplay among these factors, the relationship context, and the situational context (see Figure 1).

### 2.2. Two Different Frameworks

Theoretical perspectives on IPV have shifted from singular explanations to more comprehensive, multifactor frameworks. These frameworks propose that partner violence is not explained solely by an individual’s distorted beliefs or psychological dysfunction but instead arises from interactions between individual characteristics and environmental conditions (Stith et al., 2004). One notable example is Dutton’s nested ecological theory (Dutton, 1995). This framework identifies four levels of factors associated with individuals who perpetrate IPV and their environments. At the macrosystem level, which represents the broadest scope, the focus is on overarching cultural values and societal beliefs. The exosystem level includes the individual’s formal and informal social structures, such as friendships, workplace dynamics, support networks, and legal systems, which link the individual and their family to broader societal contexts. The microsystem level focuses on the immediate environment where abusive behaviors occur, encompassing elements such as the family unit, precursors and consequences of abuse, and relational dynamics. Finally, the ontogenetic level emphasizes the individual’s developmental history, highlighting the impact of past experiences on current relationships.

The dyadic model of partner violence (Bartholomew & Cobb, 2011) and Dutton’s nested ecological theory (Dutton, 1995) differ in several respects. While the dyadic model considers both romantic partners within the relationship, Dutton’s framework primarily focuses on the individual who perpetrates IPV. This is a primary distinction between the two models. The dyadic model emphasizes the importance of considering both partners’ characteristics and behaviors when examining the emergence, escalation, and maintenance of violence. This relational framing does not imply equivalent agency, mutuality, or shared responsibility; responsibility for a violent or coercive act lies with the person who commits it.

The two models also differ in structure. Dutton’s nested ecological theory organizes risk factors for partner violence into concentric layers, reflecting a nested systems approach in which broader levels encompass and influence inner levels. This framework focuses on the individual’s environment, categorizing risk markers as either proximal or distal to violence. In contrast, the dyadic model of partner violence emphasizes both linear and bidirectional connections, highlighting the reciprocal influence of risk factors, their mediating effects across levels, and cross-partner effects.

The dyadic model does not explicitly incorporate the broader societal and cultural contexts that shape individual behavior, relationship dynamics, and specific situations (Bartholomew & Cobb, 2011). In contrast, Dutton’s model includes the macrosystem, which encompasses the societal context, including cultural attitudes, beliefs, and laws that influence individuals’ lives (Spencer et al., 2022).

The dyadic model prioritizes individual, relational, and situational factors directly relevant to IPV, aligning with the primary focus of psychological inquiry. Bartholomew and Cobb (2011) acknowledged this exclusion as a limitation of their framework (see below for further details). In sum, the dyadic model is a more recent framework and offers a suitable approach for conceptualizing IPV because of its interpersonal, interactive, and dynamic focus. However, much of the existing literature continues to rely heavily on Dutton’s nested ecological theory as its primary framework.

Johnson’s (2008) typology offers a complementary lens by distinguishing forms of IPV according to the role of coercive control. Intimate terrorism involves violence embedded in a broader, generally asymmetric pattern of coercive control, whereas situational couple violence arises when particular conflicts escalate without a pervasive pattern of control; violent resistance refers to violence used in response to intimate terrorism. Johnson’s framework is primarily a typology of violent patterns, while the dyadic model is a multilevel process framework that organizes background and dispositional, relationship, and situational risk markers and their reciprocal influences. The frameworks therefore address different questions and can be used together: Johnson’s typology helps identify the pattern of violence, whereas the dyadic model may help explain pathways through which that pattern develops or persists. Importantly, the dyadic model should not be interpreted as assuming symmetric control, equivalent agency, or shared responsibility.

### 2.3. Areas for Improvement

The dyadic model has several limitations. In their review of the literature, Bartholomew and Cobb (2011) primarily focused on the perpetration of IPV. They argued that, given the reported prevalence of reciprocal abuse in relationships, the risk markers of IPV perpetration and victimization largely overlap. While this perspective aligns with the bidirectional nature of IPV, where violence inflicted by one partner results in victimization of the other, it reflects a broader trend in the empirical literature, which has historically placed greater emphasis on perpetration.

Evidence suggests that risk markers of IPV are often more similar than different across roles (Costa et al., 2015; Curtis et al., 2023). However, this assumption may obscure risk markers that are specific to either perpetration or victimization.

While bidirectional violence may occur in some relationships (Machado et al., 2024), it does not necessarily imply that the risk markers for perpetration and victimization are identical. Bartholomew and Cobb (2011) argued that risk markers for IPV perpetration and victimization substantially overlap. However, overlap between these sets of markers does not mean that the two experiences are interchangeable. In this review, bidirectional violence refers only to relationships in which both partners were reported to have used violent acts. The term does not imply equivalence in motive, coercive control, severity, consequences, or responsibility. Perpetration and victimization are therefore examined separately to identify shared and role-specific risk markers.

Bartholomew and Cobb (2011) primarily focused on physical rather than psychological abuse, reflecting the emphasis of the empirical literature at the time. These authors anticipated that their proposed model could also be applied to psychological abuse.

Although this framework may be applicable to different types of IPV, each form of violence may have distinct risk markers. Much of the existing literature on IPV has examined these forms simultaneously or failed to distinguish among them. Although overlap exists, this approach may fail to capture the distinctive characteristics that differentiate forms of IPV. For instance, some prior meta-analyses on risk markers for IPV aggregated effect sizes for physical IPV with those of other forms of IPV. This aggregation may introduce confounding influences, potentially compromising the accuracy of mean effect sizes associated with specific risk markers (Spencer et al., 2019a).

Finally, Bartholomew and Cobb (2011) categorized the literature on risk markers for physical IPV perpetration by men against women in heterosexual relationships within their framework. Gender was considered only when theoretical or empirical evidence suggested that it might moderate associations with IPV. They noted that observed patterns of findings tended to be consistent across gender groups and various relationship types, including same-sex relationships as well as dating, married, and cohabiting couples (Bartholomew & Cobb, 2011). While some commonalities exist, this generalization may obscure important differences.

### 2.4. Aims of the Current Review

This review builds upon and extends the work of Bartholomew and Cobb (2011), using the dyadic model of partner violence as its conceptual framework. It addresses two limitations of the original model by including risk markers for both IPV perpetration and victimization and by considering gender differences. Consistent with the original model, the present review focuses specifically on physical IPV within heterosexual relationships.

This selective review examines the literature on physical IPV, with particular emphasis on meta-analytic findings. The review seeks to categorize risk markers associated with physical IPV by aligning them with the first three levels of the dyadic model. In doing so, it provides an updated organization of risk markers for physical IPV while incorporating more recent research findings to refine and extend the model.

Finally, meta-analytic evidence may be limited for some levels of the model. This review identifies these gaps and priorities for future research on risk markers for physical IPV perpetration and victimization in heterosexual relationships.

## 3. Methodology

### 3.1. Review Design and Search

This article is a selective review and conceptual synthesis of meta-analytic evidence, rather than a systematic or structured review. It was not preregistered. Targeted electronic searches and reference-list searches were conducted between October and December 2024 to identify peer-reviewed meta-analyses concerning risk markers associated with physical intimate partner violence (IPV). Search concepts included intimate partner or domestic violence, physical aggression or abuse, risk markers or risk factors, perpetration or victimization, and meta-analysis. Because the review was not originally designed as a systematic review, the complete list of databases, database-specific search histories and syntax, initial retrieval counts, and deduplication counts were not prospectively archived and cannot now be verified. The present review did not independently apply a standardized appraisal of the methodological quality or risk of bias of the included meta-analytic reviews.

### 3.2. Scope, Eligibility, and Study Selection

Eligibility criteria were designed to preserve conceptual comparability with the original dyadic model and statistical comparability where possible. Included meta-analyses had to report physical IPV as a distinct outcome, separately from psychological, sexual, or aggregated IPV outcomes, and had to distinguish perpetration from victimization. Other forms of IPV could be examined as risk markers, provided that physical IPV remained a separately identifiable outcome. The synthesis focused on predominantly adult heterosexual relationships, consistent with the population emphasized in the original model and the role-specific and binary gender comparisons available in the source meta-analyses. Adolescent dating samples and reviews limited to highly specific populations or sociocultural contexts were treated as requiring separate developmental or contextual interpretation. These scope decisions do not imply that excluded populations are less important or that the model is necessarily inapplicable to them.

Meta-analyses were eligible for inclusion if they: (1) specifically examined physical IPV perpetration and/or victimization; (2) reported physical IPV separately from other forms of IPV; (3) analyzed multiple risk markers associated with physical IPV; (4) focused on heterosexual romantic relationships, including married or cohabiting couples; and (5) examined physical IPV among adults, excluding studies on dating violence in young adults or studies that combined childhood and/or adolescent risk factors with adult samples.

Studies were excluded if they: (1) were systematic reviews rather than meta-analyses; (2) did not provide separable physical IPV outcomes; (3) included both adult and adolescent samples; (4) included same-sex couples or combined heterosexual and same-sex couples; (5) focused exclusively on women aged 15 years and older; (6) focused exclusively on low- and middle-income countries; (7) combined effect sizes for perpetration and victimization; (8) focused on a specific population, such as individuals with traumatic brain injuries, pregnant women, or military populations; or (9) examined only a single risk marker.

The retained review archive contained 20 candidate papers that underwent full-text assessment. Eight meta-analyses met the eligibility criteria and formed the core synthesis. Twelve papers were excluded. Based on the principal reason for exclusion, five focused on childhood or developmental exposures to violence; four examined other or combined forms of IPV or context-specific evidence that did not provide separable meta-analytic estimates specific to physical IPV and IPV role; and three focused on same-sex or sexual-minority relationships. Some papers met more than one exclusion criterion and were categorized according to the principal reason for exclusion.

Table 1 provides an overview of the eight included meta-analyses.

### 3.3. Classification of Risk Markers

The present review reorganizes previously identified risk markers for physical IPV perpetration and victimization and categorizes them according to the structure of the dyadic model of partner violence (Bartholomew & Cobb, 2011). Risk markers were organized across the first three levels of the dyadic model through theory-guided conceptual mapping rather than formal coding. The first author conducted the mapping by comparing each risk marker with the definitions and examples provided by Bartholomew and Cobb (2011). Background and dispositional markers were defined as relatively stable individual characteristics or prior experiences; relationship-context markers were defined as ongoing characteristics, experiences, or interaction patterns within the relationship; and situational-context markers were defined as acute or proximal states, events, or conditions surrounding a potential episode of physical IPV. When a risk marker could plausibly fit more than one level, its operationalization in the source meta-analysis and its closest conceptual correspondence to these definitions and examples guided its placement. The corresponding author reviewed the completed mapping but did not independently repeat it. No disagreements regarding placement arose during this review; therefore, no formal adjudication procedure was required. Because one author conducted the mapping rather than multiple coders conducting it independently, no interrater reliability coefficient or other reliability check was calculated. The resulting mapping should be understood as a theory-guided application of the existing dyadic model to recent meta-analytic evidence rather than as a newly derived coding system. Consistent with the interconnected structure of the original model, some risk markers may plausibly relate to more than one level.

## 4. Background and Dispositional Risk Markers

The first level encompasses the individual background and dispositional characteristics of partners. It considers the influence of relatively stable characteristics, lived experiences, and enduring individual traits on each partner’s propensity for violence (Bartholomew & Cobb, 2011). This level is comparable to the ontogenetic level in Dutton’s model (Dutton, 1995), which emphasizes individual factors relevant to the perpetrator. However, the dyadic model extends beyond this perspective by explicitly incorporating cross-partner effects. Factors at this level may include personal beliefs and attitudes, cognitive attributions, personality vulnerabilities, negative emotionality, mental health concerns, substance use, and other characteristics directly associated with the individual.

Within the dyadic model, partners’ background and dispositional characteristics are hypothesized to interact with one another and with relationship- and situational-context processes. The meta-analytic estimates reviewed here generally assess these markers as separate associations. Accordingly, the cross-partner effects and pathways considered in this section represent theory-guided interpretations of the model rather than mechanisms directly tested in the source meta-analyses.

### 4.1. Perpetration Risk Markers

Earlier meta-analyses often focused on a single risk marker for IPV or a limited set of markers. For example, Sugarman and Frankel (1996) examined the relationship between attitudes toward violence, attitudes toward women, gender role beliefs, and both IPV perpetration and victimization. They found that positive attitudes toward violence (*r* = 0.33) and traditional gender-role beliefs about women (*r* = 0.26) were significantly associated with IPV perpetration. Given the cross-sectional nature of the data and the consistency of the findings among men, these attitudes may reflect post hoc justifications for violence rather than causal antecedents.

Stith et al. (2000) investigated the relationship between experiencing or witnessing violence in one’s family of origin and subsequent involvement in IPV. Their analysis identified effect sizes ranging from r=0.08 to r=0.35, indicating varying levels of association between childhood exposure to family violence and later involvement in IPV.

Stith et al. (2004) conducted a meta-analysis to identify risk markers for both perpetration and victimization of physical IPV. They incorporated data from 85 studies, yielding 308 distinct effect sizes, which were synthesized into composite effect sizes for 16 perpetration risk markers (see Table 1). In the present review, several markers were mapped to the background and dispositional level of the dyadic model. Moderate risk markers for men who perpetrated physical IPV against women included illicit drug use (r=0.31), attitudes condoning violence (r=0.30), traditional sex-role ideology (r=0.29), anger/hostility (r=0.26), alcohol abuse (r=0.24), and depression (r=0.23) (Stith et al., 2004).

Oram et al. (2014) conducted a systematic review and meta-analysis examining diagnosed psychiatric disorders in relation to physical violence against a partner. Their pooled estimates indicated higher odds of having ever used physical violence against a partner among men with depression (OR=2.8, 95% CI [2.5, 3.3]), generalized anxiety disorder (OR=3.2, 95% CI [2.3, 4.4]), and panic disorder (OR=2.5, 95% CI [1.7, 3.6]). Higher odds were also observed among women with depression (OR=2.4, 95% CI [2.1, 2.8]), generalized anxiety disorder (OR=2.4, 95% CI [1.9, 3.0]), and panic disorder (OR=1.9, 95% CI [1.4, 2.5]).

These estimates primarily concerned lifetime violence rather than current or prospectively observed perpetration. The authors also noted that potential confounding by substance misuse, prior violence, and other clinical and social factors could not be fully addressed; consequently, the findings do not establish a causal relationship between psychiatric disorder and IPV perpetration. Within the dyadic model, psychiatric disorders can be situated at the background and dispositional level as individual characteristics associated with IPV perpetration. However, the meta-analysis did not test whether these associations operate through emotion regulation, communication, acute stressors, or other relationship- or situational-level processes. Such cross-level pathways remain theoretical propositions requiring longitudinal dyadic investigation.

Spencer et al. (2019a) conducted a meta-analysis examining the relationship between depression, anxiety, posttraumatic stress disorder (PTSD), antisocial personality disorder (APD), borderline personality disorder (BPD), and physical IPV among men and women. The authors investigated gender as a moderating factor, recognizing that mental health disorders often manifest differently in men and women, particularly with respect to externalizing and internalizing symptoms (Tolin & Foa, 2006). They found that depression (r=0.21), anxiety (r=0.13), PTSD (r=0.21), antisocial personality disorder (r=0.27), and borderline personality disorder (r=0.34) were significant risk markers for IPV perpetration when effect sizes for men and women were combined (Spencer et al., 2019a).

Within the dyadic model, these mental health variables are situated at the background and dispositional level as risk markers associated with IPV perpetration. However, Spencer et al. (2019a) emphasized that their meta-analysis measured correlates rather than causal effects and noted that cross-sectional designs were common in this literature, preventing conclusions about whether mental health problems preceded or followed IPV. Emotional dysregulation, impulsivity, hostility, and related interpersonal processes may be theoretically relevant to these associations, but the pooled estimates did not test mediation, cross-partner effects, or causal mechanisms. These processes should therefore be treated as hypotheses for future longitudinal dyadic research rather than as explanations established by the meta-analysis.

Spencer et al. (2022) conducted a meta-analysis to identify risk markers for physical IPV perpetration among men and women in adult opposite-sex relationships. They synthesized evidence from studies published between 1980 and 2018 and compared the relative strength of 44 risk markers. The analysis identified 63 shared risk markers for physical IPV perpetration for men and women, as well as 60 risk markers unique to men who perpetrated IPV and 45 unique to women who perpetrated IPV.

The present review reorganized the risk markers identified by Spencer et al. (2022) within the framework of the dyadic model. At the background and dispositional level, key risk markers included anger (r=0.32), antisocial personality disorder (r=0.27), general mental health problems (r=0.27), narcissism (r=0.26), external locus of control (r=0.26), prior arrest (r=0.26), drug use (r=0.25), access to weapons (r=0.24), and substance use, including both drug and alcohol use (r=0.22).

Additional risk markers included childhood abuse (r=0.22), witnessing parental IPV (r=0.22), depression (r=0.22), alcohol use (r=0.21), PTSD (r=0.21), impulsivity (r=0.21), adherence to traditional gender roles (r=0.20), trauma (r=0.18), anxiety (r=0.16), stress (r=0.16), and physical health problems (r=0.11). Insecure attachment styles were also significantly associated with IPV perpetration, including anxious attachment (r=0.16), avoidant attachment (r=0.13), and disorganized attachment (r=0.11).

Based on combined effect sizes for men and women, higher income (r=−0.17), higher self-esteem (r=−0.14), higher education (r=−0.14), secure attachment (r=−0.11), older age (r=−0.10), employment (r=−0.07), and religiosity (r=−0.07) were inversely associated with physical IPV perpetration (Spencer et al., 2022). These estimates identify inverse associations but do not establish that these variables prevent IPV or interrupt escalation.

**Summary.** A broad range of variables associated with physical IPV perpetration were mapped to the background and dispositional level of the dyadic model. The reviewed meta-analyses generally estimated these associations separately and did not test interactions between partners or pathways across levels. Anger or hostility, substance use, mental health variables, and prior exposure to violence were among the recurrent risk markers, whereas income, self-esteem, education, secure attachment, age, employment, and religiosity showed inverse associations in Spencer et al. (2022). Within the dyadic model, these findings identify background and dispositional characteristics that can be examined jointly with relationship- and situational-context markers in future longitudinal dyadic research.

### 4.2. Victimization Risk Markers

Stith et al. (2004) identified depression (r=0.28) and fear of partner violence (r=0.27) as moderate risk markers for women’s victimization. Alcohol abuse (r=0.13) was identified as a smaller risk marker for women’s victimization. Stith et al. (2004) noted that depression and fear may be consequences of partner violence rather than causes. Within the dyadic model, these variables are nevertheless situated at the background and dispositional level because they describe the individual’s psychological context, which may both shape and be shaped by ongoing relationship experiences.

Spencer et al. (2019b) conducted a meta-analysis investigating risk markers associated with physical IPV victimization. Using data from 391 studies that produced 1731 effect sizes, their analysis evaluated 50 risk markers in women and 28 risk markers in men. The strength of each marker was assessed for men and women, and gender was examined as a moderator for 28 markers.

The present review reorganized the identified risk markers for physical IPV victimization among men and women within the framework of the dyadic model of partner violence. When data for men and women were combined, PTSD (r=0.34), depression (r=0.25), anxiety (r=0.19), antisocial personality disorder (r=0.18), and borderline personality disorder (r=0.19) emerged as significant risk markers for IPV victimization (Spencer et al., 2019a). Within the dyadic model, these mental health markers form part of the psychological context that each partner brings to the relationship, while some may also reflect consequences of prior or ongoing victimization. Their relevance therefore lies in how they may be embedded within relationship processes over time, rather than in treating them as isolated explanations of victimization.

For men, risk markers included a history of abuse in past relationships (r=0.49), PTSD (r=0.29), borderline personality disorder (r=0.27), stress (r=0.24), anger (r=0.22), antisocial personality disorder (r=0.22), drug use (r=0.21), depression (r=0.18), witnessing IPV in the family of origin (r=0.17), and anxiety (r=0.16). Additional risk markers included experiencing childhood abuse within the family (r=0.15), alcohol use (r=0.14), and impulsivity (r=0.13) (Spencer et al., 2019b). Prior trauma, personality disorders, and chronic stress responses were mapped to the background and dispositional level because they reflect previous experiences or relatively enduring aspects of psychological functioning. Within the dyadic model, these markers may intersect with the partner’s characteristics and current relationship processes; their meaning therefore depends on the broader relational context.

For men, older age (r=−0.13) was inversely associated with IPV victimization. Educational attainment, employment status, and general mental health problems were not significantly associated with IPV victimization (Spencer et al., 2019b).

Spencer et al. (2019b) also identified several risk markers for IPV victimization among women, including PTSD (r=0.34), fear (r=0.29), depression (r=0.29), a history of abuse in past relationships (r=0.27), drug use (r=0.25), antisocial personality disorder (r=0.23), and childhood abuse within the family of origin (r=0.23).

Additional risk markers among women included substance use (r=0.22), alcohol use (r=0.21), anger (r=0.21), witnessing IPV in the family of origin (r=0.20), borderline personality disorder (r=0.20), anxious attachment (r=0.20), approval of violence (r=0.17), stress (r=0.16), mental health problems (r=0.14), adherence to traditional gender roles (r=0.08), and impulsivity (r=0.07). Several of these markers, particularly PTSD, fear, depression, and substance use, may reflect consequences of victimization as well as pre-existing characteristics. Within the dyadic model, they should be considered in conjunction with partner behavior, coercive control, prior violence, and the broader relationship context.

Higher education (r=−0.09) and older age (r=−0.02) were inversely associated with IPV victimization among women, although these effects were relatively small. Several theoretically relevant variables were not significantly associated with IPV victimization, including secure attachment, avoidant attachment, physical health, self-blame, self-esteem, trauma, prior arrest, religiosity, and employment (Spencer et al., 2019b). These findings underscore the complexity of victimization processes: not all theoretically relevant variables were significantly associated with IPV victimization.

**Summary.** Mental health difficulties, personality disorders, histories of abuse, substance use, anger, and impulsivity were recurrent background and dispositional markers associated with physical IPV victimization. Within the dyadic model, these markers characterize the psychological and experiential context that each partner brings to the relationship. They are hypothesized to interact with relationship-level processes and may also be shaped by prior or ongoing victimization. Their interpretation therefore requires attention to partner behavior, coercive control, prior violence, and situational context. The overlap of markers between men and women underscores the value of examining both partners and multiple model levels over time, while differences in effect size indicate that association strength varies between men and women.

### 4.3. Gender Differences

Whether IPV patterns and associated risk markers differ between men and women remains a subject of ongoing debate (Spencer et al., 2016). This section examines gender differences in risk markers for IPV perpetration and victimization, with particular attention to factors classified within the background and dispositional level of the dyadic model.

Cafferky et al. (2018) conducted a meta-analysis examining the association between substance use, specifically alcohol and drug use, and IPV. The study compared the associations of overall substance use, alcohol use, and drug use with IPV perpetration and victimization between men and women. Overall substance use was more strongly associated with IPV perpetration among men (r=0.23) than among women (r=0.17), indicating a stronger association among men. Similarly, alcohol use demonstrated a stronger association among men (r=0.22) than among women (r=0.15). However, no gender differences were observed in the association between drug use and IPV perpetration. Substance use was more strongly associated with IPV victimization among women (r=0.21) than among men (r=0.17). No gender differences were identified in the independent associations between alcohol use or drug use and IPV victimization (Cafferky et al., 2018).

Spencer et al. (2016) conducted a meta-analysis examining gender differences in risk markers for physical IPV perpetration. They compared 60 distinct risk markers associated with IPV perpetration in men and women. Risk markers classified within the background and dispositional level included age, education, income, employment status, depression, anxiety, anger, stress, trauma, self-esteem, PTSD, antisocial personality disorder, borderline personality disorder, general mental health problems, drug use, alcohol-related difficulties, physical health, history of spousal abuse, locus of control, approval of violence, religiosity, coping skills, impulsivity, beliefs in male privilege, prior arrest, attachment styles, and attributional tendencies such as self-blame and blaming others.

The analysis also included family-of-origin variables, such as witnessing parental IPV, observing violence perpetrated by either parent, and experiencing childhood physical abuse. Of the 60 risk markers examined, only three demonstrated significant gender differences. These included witnessing IPV, experiencing childhood abuse, and alcohol-related difficulties. When witnessing parental IPV and experiencing childhood abuse were analyzed separately, gender did not significantly moderate the associations. However, when combined into a single indicator of family-of-origin violence, gender significantly moderated the association. This combined effect was stronger for men (r=0.25) than for women (r=0.19), indicating that the association between family-of-origin violence and IPV perpetration was stronger among men (Spencer et al., 2016).

Gender also moderated the association between alcohol use and IPV perpetration. The effect size for alcohol-related difficulties among men (r=0.22) was significantly larger than among women (r=0.15), indicating a stronger association between alcohol use and IPV perpetration among men (Spencer et al., 2016).

A subsequent meta-analysis found no significant gender differences in the association between mental health disorders and IPV perpetration. However, depression emerged as a stronger correlate of IPV victimization among women (r=0.28) than among men (r=0.18) (Spencer et al., 2019a). Similarly, Oram et al. (2014) found higher odds of lifetime physical IPV perpetration among both men and women with psychiatric disorders than among comparison groups, with larger estimates for men. Psychiatric disorders were also associated with IPV victimization among both men and women in other studies, with stronger associations among women (Khalifeh & Dean, 2010; Oram et al., 2014; Teplin et al., 2005).

These findings suggest that gender differences in risk markers for IPV are generally subtle rather than dramatic. Differences are often reflected in the relative strength of associations rather than the presence or absence of specific risk markers. The present section focuses on such differences in risk profiles, whereas gender differences in the pattern and direction of violence are discussed in subsequent sections.

Spencer et al. (2022) examined 44 risk markers for IPV perpetration and compared associations between men and women. When effect sizes were analyzed separately, nine risk markers differed significantly between men and women who perpetrated IPV. Alcohol use (r=0.22), combined substance use (r=0.22), childhood abuse (r=0.24), and witnessing parental IPV (r=0.25) emerged as stronger risk markers for IPV perpetration among men.

An internal locus of control, defined as the belief that individuals are responsible for their own actions, showed the strongest inverse association with IPV perpetration among men (r=−0.30). Religiosity (r=−0.09) and higher educational attainment (r=−0.15) also showed stronger inverse associations among men than among women. Among women, general mental health problems (r=0.15) and approval of violence (r=0.14) were not significantly associated with physical IPV perpetration. The inverse associations involving higher self-esteem (r=−0.11), employment (r=−0.05), and religiosity (r=−0.02) were also not statistically significant among women (Spencer et al., 2022).

In their meta-analysis of physical IPV victimization, Spencer et al. (2019b) examined 28 risk markers and identified five that significantly differed by gender. Depression (r=0.29), alcohol use (r=0.19), and childhood abuse within the family of origin (r=0.23) emerged as stronger risk markers for victimization among women. Conversely, older age showed a stronger inverse association with IPV victimization among men (r=−0.14).

**Summary.** Gender differences were generally reflected in the relative strength of associations rather than in the presence or absence of particular risk markers. Although the source meta-analyses reported statistically significant gender differences for some associations, they did not establish whether those differences were clinically significant. We therefore do not characterize these differences as meaningful. Among the reported differences, associations of alcohol use and family-of-origin violence with IPV perpetration were stronger among men, whereas associations of depression and childhood abuse with IPV victimization were stronger among women. These findings support consideration of both shared and gender-differentiated associations within the background and dispositional level of the dyadic model. Within the model, these markers are hypothesized to interact with relationship-context processes, but such cross-level pathways require testing through longitudinal dyadic research.

### 4.4. Differences Between Perpetration and Victimization

Research has linked psychiatric disorders with both IPV perpetration and victimization (Jonas et al., 2014; Trevillion et al., 2012). Spencer et al. (2019a) identified differences in the strength of associations between mental health variables and the two IPV roles. PTSD (r=0.34) and anxiety (r=0.19) were more strongly associated with IPV victimization, whereas borderline personality disorder (r=0.34) and antisocial personality disorder (r=0.27) were more strongly associated with IPV perpetration. No significant role difference was observed for depression. Spencer et al. (2019a) also examined whether the relative strength of these associations differed by gender. No significant differences between perpetration and victimization were identified among men. Among women, PTSD was more strongly associated with victimization (r=0.35) than perpetration (r=0.17), whereas borderline personality disorder was more strongly associated with perpetration (r=0.35) than victimization (r=0.20).

These findings indicate that some mental health associations differ according to IPV role and gender. Cafferky et al. (2018) similarly found that overall substance use, alcohol use, and drug use were associated with both IPV perpetration and victimization, with mean effect sizes ranging from r=0.18 to r=0.23. The associations of alcohol use (r=0.20) and drug use (r=0.23) with perpetration did not differ significantly. For victimization, however, drug use (r=0.23) showed a stronger association than alcohol use (r=0.17). Problematic alcohol-use measures were more strongly associated with victimization than alcohol-consumption measures, whereas the two measurement types showed similar associations with perpetration. Problematic drug-use measures were more strongly associated with perpetration than drug-consumption measures. No significant differences were found among drug types or between stimulants and nonstimulants for either IPV role. These findings indicate that the strength of substance-use associations varies according to IPV role and how substance use is measured.

**Summary.** Table 2 summarizes the risk markers mapped to the background and dispositional level. Mental health and substance-use variables were associated with both IPV perpetration and victimization, but the relative strength of these associations differed by role. PTSD and anxiety were more strongly associated with victimization, whereas borderline and antisocial personality disorders were more strongly associated with perpetration. Among women, PTSD was more strongly associated with victimization and borderline personality disorder with perpetration; no significant role differences were identified among men. Alcohol and drug use were similarly associated with perpetration, whereas drug use was more strongly associated than alcohol use with victimization. These patterns distinguish relative strengths of association rather than causal determinants of perpetration or victimization. Within the dyadic model, they support examining background and dispositional markers in conjunction with both partners’ characteristics and the relationship and situational contexts.

## 5. Relationship Context

The relationship context represents the second level of the dyadic model and concerns ongoing characteristics, experiences, and interaction patterns within the current relationship. These processes emerge from the interplay between partners and their enduring characteristics (Bartholomew & Cobb, 2011). Across the included meta-analytic reviews, some of the largest associations with physical IPV occurred at this level, particularly for prior or concurrent violence, injury, emotional abuse, and relationship dissatisfaction (Spencer et al., 2019b, 2022). This pattern was evident for both perpetration and victimization. The source meta-analyses generally estimated these relationship-context markers as separate associations; therefore, reciprocal sequences and cross-level pathways discussed in this section represent theory-guided interpretations rather than processes directly tested in the pooled evidence.

### 5.1. Perpetration Risk Markers

Within the relationship context, the relevance of each partner’s characteristics lies partly in how partners negotiate power, intimacy, and conflict in their daily interactions. Chronic patterns of emotional aggression or coercive control can become persistent features of a relationship. Stith et al. (2004) reported that emotional abuse had the largest association with physical IPV perpetration among men (r=0.49). A history of physical abuse toward the current partner showed a moderate association with current physical IPV perpetration (r=0.24). Marital satisfaction was inversely associated with perpetration among both men (r=−0.30) and women (r=−0.25). Together, these findings situate physical IPV perpetration within relational contexts marked by emotional abuse, prior violence, and relationship dissatisfaction.

Stith et al. (2008) reported a moderate inverse association between marital satisfaction and physical IPV perpetration (r=−0.27). Marital satisfaction and marital discord showed associations of equivalent magnitude. Thus, lower satisfaction and greater discord were associated with perpetration, but the pooled estimates did not establish that either variable caused physical aggression. The association varied by the gender of the person who perpetrated IPV, the gender of the person who experienced IPV, and the direction of violence; these findings are discussed below.

Spencer et al. (2022) reported that the largest relationship-context associations with perpetration involved prior injury to a partner (r=0.58), emotional IPV perpetration (r=0.53), and physical IPV victimization (r=0.52). Additional markers included stalking perpetration, assessed among men (r=0.47); emotional IPV victimization (r=0.44); sexual IPV victimization (r=0.44); and prior physical IPV perpetration (r=0.42). These findings indicate that physical IPV perpetration often co-occurs with broader patterns of aggression, coercion, victimization, and harm within the relationship.

Other relationship-context markers included demand/withdraw interaction patterns (r=0.37), controlling behavior (r=0.30), and greater perpetrator power within the relationship (r=0.18). Relationship satisfaction (r=−0.25), communication skills (r=−0.24), coping skills (r=−0.20), conflict-resolution skills (r=−0.17), length of cohabitation (r=−0.16), relationship duration (r=−0.11), and empathy, assessed among men (r=−0.14), were inversely associated with IPV perpetration. Marital status was not significantly associated with perpetration (Spencer et al., 2022). Within the dyadic model, these variables characterize ongoing relationship processes and resources; the inverse associations do not by themselves establish protective mechanisms.

**Summary.** Relationship-context markers showed some of the largest associations with physical IPV perpetration. Prior or concurrent aggression, injury, emotional abuse, physical or sexual victimization, stalking, demand/withdraw interaction, controlling behavior, and power imbalance were positively associated with perpetration, whereas relationship satisfaction, communication, coping, conflict resolution, relationship duration, and empathy showed inverse associations. This pattern suggests that physical IPV perpetration is frequently embedded within broader relational contexts of aggression, coercion, harm, and dissatisfaction. Within the dyadic model, these findings support attention to ongoing relationship processes, while the temporal ordering and reciprocal pathways among them require longitudinal dyadic investigation.

### 5.2. Victimization Risk Markers

At the relationship-context level, partners’ ongoing interpersonal exchanges and conflict patterns are dynamic and differ across couples. Within the dyadic model, these processes form part of the relational context in which physical IPV victimization occurs. Spencer et al. (2019b) examined 50 potential risk markers among women and 28 among men who experienced IPV. They also tested whether the strength of 28 associations differed between men and women. The present review maps these markers onto the dyadic model.

The largest associations with victimization for both men and women were concentrated at the relationship-context level, situating victimization within ongoing relationship patterns rather than as an isolated event. Prior violence within the relationship was a prominent marker for both men and women who experienced IPV. Among men who experienced IPV, the strongest risk markers of IPV victimization included having previously sustained injuries from the perpetrator (r=0.64), experiencing emotional abuse (r=0.53), a history of perpetrating IPV (r=0.49), engaging in emotional abuse toward a partner (r=0.42), and relationship dissatisfaction (r=0.23). Neither sexual IPV victimization nor perpetrator power dynamics emerged as risk markers for men’s physical victimization. Relationship characteristics such as marital status and relationship length showed no association with men’s risk of victimization (Spencer et al., 2019b).

These findings are consistent with possible overlap between perpetration and victimization; however, the pooled associations do not establish a reciprocal or cyclical sequence between partners. For women, the relationship context also contained several of the largest associations with IPV victimization. The strongest risk markers for women’s physical victimization included previous perpetration (r=0.56), previous injury caused by the perpetrator (r=0.54), emotional victimization (r=0.51), sexual victimization (r=0.44), emotional perpetration (r=0.41), and stalking victimization (r=0.40). These associations indicate that multiple forms of aggression and harm co-occurred with physical IPV victimization. They do not establish that one act triggered another or that escalation occurred over time. Additional markers included demand/withdraw patterns (r=0.32), perpetrator’s controlling behaviors (r=0.31), perpetrator’s power (r=0.25), and relationship dissatisfaction (r=0.27). For women, communication skills were inversely associated with victimization (r=−0.17). However, neither relationship duration (r=−0.04) nor conflict-resolution skills (r=−0.07) were associated with IPV victimization among women (Spencer et al., 2019b).

**Summary.** Prior violence, emotional abuse, and relationship dissatisfaction were among the strongest relationship-context markers associated with physical IPV victimization for both men and women. For women, sexual and stalking victimization, controlling behavior, partner power, and demand/withdraw patterns were additional relationship-context markers, while communication skills showed an inverse association. Overall, these findings situate victimization within a broader relational context without implying that the observed markers determine the occurrence or course of violence.

### 5.3. Gender Differences

Within the relationship context, this section examines whether the strength of associations between relational risk markers and IPV differs between men and women. Stith et al. (2008) reported an inverse association between marital satisfaction and IPV perpetration overall (r=−0.27), with a somewhat stronger association for men (r=−0.28) than women (r=−0.21). The association between marital satisfaction and IPV was stronger for victimization (r=−0.41) than perpetration (r=−0.27), and the victimization association was stronger for women (r=−0.41) than men (r=−0.30). These findings indicate differences in association strength across IPV role and gender.

Spencer et al. (2016) examined whether gender moderated associations between physical IPV perpetration and relationship-context markers, including marital status and relationship duration; relationship satisfaction and distress; communication, conflict-resolution, and demand/withdraw patterns; psychological abuse; prior violence or victimization involving the current partner; and indicators of severity such as weapon use, property destruction, and injury. Gender did not moderate most of these associations. For instance, a history of emotional abuse showed a moderate-to-large association with perpetration among both men (r=0.49) and women (r=0.48). Similarly, relationship satisfaction showed a small negative correlation with perpetration, with identical effect sizes for both men and women (r=−0.22).

These findings indicate that emotional abuse and relationship satisfaction were associated with IPV perpetration to a similar degree among men and women. Similarity in association strength, however, does not establish that the underlying relational processes are identical for men and women. A gender difference emerged in the association between demand/withdraw communication patterns and IPV perpetration. The effect was stronger when men assumed the demanding role and women the withdrawing role (r=0.41). In contrast, when the roles were reversed, with women demanding and men withdrawing, the association was notably weaker (r=0.16) (Spencer et al., 2016). Within the dyadic model, this finding highlights the importance of considering which partner occupies the demanding and withdrawing roles. However, the meta-analysis did not test the social or psychological mechanisms underlying the difference in association strength.

Spencer et al. (2019b) identified significant gender differences in 5 out of 28 examined risk markers for IPV victimization. In the present review, one of these markers was categorized at the relationship-context level. Sexual IPV victimization was associated with physical IPV victimization among women (r=0.44) but not among men. This gender difference indicates greater overlap between sexual and physical IPV victimization among women; its interpretation should consider coercive control and broader patterns of abuse rather than assume that one form necessarily precedes or escalates into the other (Johnson, 2008). Spencer et al. (2022) found that male partners’ controlling behaviors were not significantly associated with women’s IPV perpetration. Relationship duration and marital status were also not significantly associated with women’s IPV perpetration, whereas conflict-resolution skills showed a stronger inverse association with perpetration among women than among men. The association between demand/withdraw patterns and IPV perpetration was stronger among men than among women.

**Summary.** Table 3 displays the risk markers categorized at the relationship-context level. Gender moderated some associations, including those involving marital satisfaction and demand/withdraw patterns, whereas emotional abuse and relationship satisfaction were associated with IPV perpetration to similar degrees among men and women. Sexual IPV victimization was associated with physical IPV victimization among women but not among men; this finding suggests overlap between forms of victimization rather than established temporal escalation. Overall, the findings show both shared and gender-differentiated relationship-context associations. They support the dyadic model’s emphasis on relational processes while indicating that similarities in association strength do not necessarily imply identical underlying processes for men and women.

## 6. Situational Context

Within the dyadic model, the situational context refers to acute or proximal states, events, and conditions surrounding an episode of IPV. Bartholomew and Cobb (2011) proposed that arguments, perceived threats, negative emotions, jealousy, rejection, infidelity, and competing needs for closeness and autonomy may shape how conflict unfolds in the immediate context. Applying the dyadic model as an organizing lens, the present review maps risk markers reported in the included meta-analyses onto the situational-context level according to their conceptual correspondence with these definitions and examples. This mapping identifies recent meta-analytic findings relevant to the immediate-context component of the model and distinguishes them from more enduring background and dispositional or relationship-context markers.

The situational-context section is less extensive than the sections addressing the other two model levels because the included meta-analytic reviews reported fewer risk markers that could be mapped to this level. Consistent with the scope of the present review, the mapping was limited to risk markers reported in those source reviews. This relative scarcity therefore reflects a gap in the eligible meta-analytic evidence reviewed here. Some constructs may nevertheless represent either acute states or more enduring individual or relationship conditions, depending on how they were operationalized. Their placement is therefore theory-guided and provisional rather than evidence that each marker functions as an immediate trigger.

### 6.1. Perpetration Risk Markers

Stith et al. (2004) reported that jealousy showed a small association with physical IPV perpetration by men (r=0.17), whereas career and life stress showed a moderate association (r=0.26). Sexual coercion showed a comparatively large association with current physical IPV perpetration (r=0.45).

Spencer et al. (2022) reported additional risk markers that were mapped to the situational-context level. Using combined effect sizes for men and women, threatening to harm one’s partner (r=0.49), verbal arguments (r=0.43), violence toward non-family members (r=0.28), and jealousy (r=0.24) were associated with IPV perpetration. Smaller associations were observed for physical abuse of one’s own children (r=0.17), financial stress (r=0.11), and number of children (r=0.08). Infidelity (r=0.22) and self-directed threats (r=0.10) were examined only among men who perpetrated IPV, so gender comparisons were unavailable.

**Summary.** The reviewed evidence identified several variables associated with IPV perpetration that were provisionally mapped to the situational-context level, including jealousy, life stress, threats, verbal arguments, sexual coercion, and infidelity. The strongest associations involved threatening to harm a partner, sexual coercion, and verbal arguments. Infidelity and self-directed threats were examined only among men, so gender comparisons were unavailable. From a dyadic perspective, these findings identify acute conditions that may intersect with background predispositions and chronic relational strains in the context surrounding physical IPV.

### 6.2. Victimization Risk Markers

At the situational-context level, conditions associated with IPV victimization are considered alongside chronic individual vulnerabilities and relationship patterns. In their review, Stith et al. (2004) identified one significant risk marker for IPV victimization. This meta-analysis found that women’s IPV perpetration against their partners was associated with women’s IPV victimization (r=0.41). The pooled estimate does not establish whether retaliation, reciprocal aggression, or escalation explains this association. Spencer et al. (2019b) reported no markers of physical IPV victimization among men that could be mapped to the situational-context level. The available literature contains limited evidence concerning situational-context risk markers for IPV victimization among men.

Incorporating these observations into the model highlights the need for more focused inquiry into situational-context markers associated with IPV victimization among men. Examining whether men and women share similar or distinct situational-context markers is an important direction for future research. The meta-analysis reported several markers positively associated with IPV victimization among women that could be mapped to this level. These markers, with effect sizes ranging from r=0.07 to r=0.39, included self-harm threats (r=0.39), perpetrator’s jealousy (r=0.23), separation (r=0.21), financial stress (r=0.15), accusations of infidelity (r=0.14), and number of children (r=0.07). Social support was not significantly associated with IPV victimization for either group (Spencer et al., 2019b).

**Summary.** These findings illustrate that situational-context risk markers for IPV victimization vary in the strength of their associations. Among women, IPV perpetration against their partners and self-harm threats showed the strongest associations with victimization. Jealousy, separation, and financial stress showed smaller positive associations with victimization.

### 6.3. Gender Differences

Available evidence identified different infidelity-related risk markers in gender-specific analyses. For men, infidelity was associated with IPV perpetration (Spencer et al., 2022), whereas for women, being accused of infidelity was associated with IPV victimization (Spencer et al., 2019b). These findings suggest that infidelity-related experiences may be relevant to different IPV roles, although the source analyses did not directly compare the same marker between men and women.

In the previously discussed meta-analysis by Spencer et al. (2016), researchers identified three risk markers with significant gender differences. However, none of these gender-specific risk markers could be mapped to the situational-context level. These findings provide limited evidence regarding gender differences at the situational-context level, particularly because research on situational risk markers remains limited. Spencer et al. (2016) examined several risk markers for IPV perpetration that can be mapped to the situational-context level. These contextual risk markers, for which no significant gender differences were reported, included social support, physical violence toward one’s children, financial stress, violence toward others outside one’s immediate family, verbal arguments, forced sex, and number of children. Despite examining these factors, the authors did not report the corresponding effect sizes for them because the gender differences were nonsignificant (Spencer et al., 2016). In a later analysis, number of children was not significantly associated with physical IPV perpetration among women. Additionally, physical abuse of one’s children showed a stronger association with IPV perpetration among men than among women (Spencer et al., 2022).

**Summary.** Table 4 displays the placement of risk markers identified within the situational-context level. Overall, relatively few situational-context markers were directly compared between men and women, limiting conclusions about gender differences at this level. Within the dyadic model, the situational context can be conceptualized as a “convergence point” at which personal and relational vulnerabilities intersect with acute conditions surrounding IPV. The reviewed associations provide a basis for mapping these markers to this level, but they do not establish an immediate temporal or causal relation between these markers and violent acts.

## 7. Discussion

Taken together, the reviewed evidence was unevenly distributed across the three levels of the dyadic model. The relationship-context level was the most consistently represented and contained several of the largest associations across perpetration and victimization, particularly those involving prior or concurrent IPV, injury, emotional abuse, sexual or stalking victimization, and relationship dissatisfaction. Background and dispositional markers, including anger or hostility, substance use, mental health conditions, and prior exposure to violence, were more numerous but generally showed smaller and more heterogeneous associations.

The comparatively larger relationship-context associations may partly reflect their closer conceptual and, in some cases, temporal proximity to physical IPV. Several of the strongest markers, such as prior or concurrent IPV, injury, emotional abuse, and other forms of victimization, also represent ongoing or closely related manifestations of aggression and harm within the same relationship. By contrast, background and dispositional markers are more distal and heterogeneous and may be associated with IPV through intervening relationship- or situational-context processes.

Differences in effect-size magnitude should therefore not be interpreted as direct evidence that one level or marker has greater causal importance. Because the source meta-analyses did not provide validated thresholds for clinical significance, the effect sizes are interpreted comparatively and in relation to their context, severity, and consequences rather than as direct indicators of clinical importance.

The situational-context level contained fewer eligible markers, reflecting a relative gap in the meta-analytic evidence reviewed here. The evidence extends the original model by organizing recent findings for perpetration and victimization separately while also demonstrating substantial overlap between the sets of markers associated with the two roles. In the binary gender comparisons available in the source reviews, many associations were similar in direction and magnitude, although a smaller subset differed in strength. These findings support the utility of the dyadic model as a conceptual framework for organizing current evidence.

However, the included meta-analyses generally estimated risk markers separately and did not directly test actor and partner effects, mediation across levels, feedback processes, or within-couple change; therefore, the present synthesis does not constitute empirical validation of the model’s integrated temporal or causal pathways. The revised dyadic framework should therefore be regarded as a theory-guided conceptual organization of the available meta-analytic associations, rather than as an empirically validated integrated model.

Similarity in the magnitude of selected risk marker associations should not be interpreted as evidence that IPV has equivalent meanings, contexts, or consequences for men and women. Effect sizes based primarily on the occurrence of physical acts may not capture motive, coercive control, fear, chronicity, severity, injury, sexual violence, or constraints on safety and leaving. Broader literature indicates that women, on average, experience greater injury, fear, sexual victimization, and posttraumatic consequences following IPV, patterns that should be understood in relation to gendered power and control (Caldwell et al., 2012).

At the same time, men can experience serious IPV victimization, and group-level patterns should not be used to minimize an individual victim’s experience. Johnson’s (2008) typology further illustrates that apparent gender symmetry may vary according to the pattern of violence: situational couple violence may show greater symmetry in the occurrence of violent acts, whereas intimate terrorism is characterized by a broader and generally asymmetric pattern of coercive control. Gender comparisons should therefore consider power, control, motive, fear, injury, severity, and consequences alongside the magnitude of statistical associations.

Against this evidentiary background, the present review classified the most salient risk markers of physical IPV identified in previous meta-analyses within the framework of the dyadic model of partner violence (Bartholomew & Cobb, 2011). This review updates the model by incorporating recent evidence, examining differences across gender groups and IPV roles, and identifying gaps requiring further investigation. Although the dyadic model proposes that perpetration and victimization roles may overlap and that strict role distinctions may sometimes be redundant, meta-analytic findings indicate subtle differences between their associated risk markers. The dyadic model offers a useful conceptual framework with considerable potential for advancing IPV research. However, the reviewed meta-analyses suggest that the model remains underused as an organizing framework.

Notably, none of the meta-analyses reviewed used the dyadic model or acknowledged its relevance to the literature. In these studies, Dutton’s (1995) nested ecological model was used consistently as an organizing framework. This reliance may limit how the IPV literature conceptualizes partner violence as a relational and deeply interpersonal phenomenon. A dynamic interpersonal model may therefore offer a particularly relevant lens for examining IPV as a context-dependent relational phenomenon. Dutton’s model, while valuable, primarily focuses on one individual at a time, most often the person who perpetrates IPV, and gives less explicit attention to relational dynamics between partners. It also gives limited attention to interactions between levels and associations among risk markers across levels.

One of the core premises of the dyadic model is that risk markers do not simply accumulate in isolation; rather, they may interact within the context of a relationship. The source meta-analyses generally examined risk markers separately and therefore did not directly test this proposed interplay. Although methodologically ambitious, future research could employ the dyadic model as a central theoretical and analytic framework for examining multiple risk markers simultaneously.

The dyadic model is a flexible framework that can accommodate variation across individuals, couples, relationships, and situations. Couples differ in their relational dynamics, and pathways from mild verbal conflict to severe physical violence may vary across relationships. An individual’s responses may also vary across romantic partners and relationships. Interpreting reports of bidirectional violence requires attention to both partners’ characteristics and behaviors and to the relationship context, while maintaining individual accountability for violent acts. The dyadic model is used here to understand relational processes and risk contexts, not to allocate blame or justify violence. Dyadic conceptualization does not assume that both partners contribute equally or that responsibility for violence is shared. Reciprocal escalation may characterize some cases of situational couple violence; by contrast, intimate terrorism is defined by an asymmetric pattern of coercive control (Johnson, 2008). In such cases, a victim’s resistance or constrained responses should not be treated as equivalent to the perpetrator’s controlling violence. Application of the dyadic model therefore requires attention to coercive control, fear, motive, self-protection or violent resistance, severity, injury, consequences, and directionality, with victim safety and perpetrator accountability remaining central.

### 7.1. Future Directions

Future research should use longitudinal dyadic designs that assess both partners repeatedly across the background and dispositional, relationship, and situational levels. Dyadic multilevel or intensive-longitudinal models can distinguish actor effects, partner effects, and reciprocal within-couple change, while event-based or ecological momentary assessment may capture proximal escalation processes (Rodriguez et al., 2017). Measurement should also be harmonized across studies: researchers should distinguish perpetration from victimization and separately assess the frequency, severity, context, motivation, and directionality of violent acts. Where safe and feasible, reports from both partners should be obtained, and measurement invariance should be evaluated across gender groups, cultural groups, and relationship types. Future studies should use the dyadic model more extensively to examine risk markers associated with IPV. This model offers a versatile framework that can be applied across diverse types of violence, relationships, and contexts. Research remains limited on same-sex and other 2SLGBTQ+ relationships, women’s IPV perpetration against men, and men’s IPV victimization. Many of the meta-analyses reviewed were unable to identify enough effect sizes to analyze risk markers of interest within these populations.

Future IPV research would benefit from studies that assess a broader range of potential risk markers, collect data from both partners across multiple model levels, and use advanced analytic techniques. Single-case designs could incorporate case formulations tailored to each couple’s dynamics. Finally, there may be no single explanation for why emotional, sexual, or physical abuse develops or becomes recurrent within some intimate relationships. Although general patterns derived from research are informative, a “one-size-fits-all” approach may be inadequate for capturing the dyadic nature of IPV.

Further theoretical integration may also be useful. Concepts from traditional interpersonal theory (Horowitz & Strack, 2011; Kiesler, 1983) could be adapted and applied to the many IPV risk markers that emerged as important in the present review. The focus could be on examining whether and how risk markers are associated with particular cognitions and behaviors in unfolding IPV situations.

For example, a central concept in traditional interpersonal theory is “parataxic distortions” (Andersen & Miranda, 2006; Carson, 1982). Past experiences and current beliefs shape how individuals interpret other people and situations. In interpersonal theory, readily activated distortions that extend beyond available information may be amplified among individuals with interpersonal or psychological difficulties, resulting in rigid or extreme responses that are only weakly grounded in the present context. Subtle interpersonal cues may activate perceptual distortions and corresponding reactions, although these processes have not been established for the IPV risk markers reviewed here. Following general interpersonal theory, different IPV risk markers may involve distinctive forms of parataxic distortion. Future research could examine whether these processes occur, how they unfold for different IPV risk markers, and whether combinations of markers interact in shaping interpersonal perceptions and responses.

Interpersonal theory also proposes that partners’ behaviors unfold in recurring, partly predictable sequences (Kiesler, 1983). In this account, hostility tends to elicit hostility, affectionate behavior tends to elicit warmth, dominance tends to elicit submission, and submission tends to elicit dominance. The evidence for these specific rules is mixed, especially with regard to dominance and submissiveness. More generally, dyadic behavior may exhibit recurring sequences in which one partner’s behavior increases the likelihood of particular responses from the other. Such order, or “grammar,” in IPV behavior sequences may not be evident in immediate A–B–A–B–A exchanges. Pivotal events, including behaviors associated with parataxic distortions, may produce qualitative shifts that make the unfolding sequences more complex than predicted by simple pairwise stimulus–response principles. IPV relationships may involve combinations of interaction patterns, including recurring behavioral sequences that warrant direct empirical investigation. Future research could examine how known IPV risk markers are embedded within repetitive and potentially complex behavioral sequences in relationships characterized by persistent hostility.

### 7.2. Diversity and Generalizability Considerations

The source meta-analyses generally used binary categories described as male/female or men/women, often referring to these comparisons as gender differences, without consistently specifying how sex and gender were operationalized. The present review therefore retains the source-reported categories as men and women while acknowledging this limitation. Accordingly, the observed group differences cannot be attributed specifically to biological sex or to gender-related social processes on the basis of the available evidence.

The focused eligibility criteria constrain the generalizability of this synthesis. The absence of same-sex couples, sexual- and gender-minority people, adolescents and young adults, low- and middle-income settings, and population-specific contexts from the core synthesis partly reflects the review design and scope. Future work should evaluate whether and how the dyadic model of partner violence requires adaptation for these populations and contexts. Within the eligible evidence set, research concerning culturally diverse populations and IPV victimization among men remained limited. Consequently, the applicability of current models to non-Western or collectivist cultural contexts, as well as to low-income or marginalized populations, remains uncertain. These limitations suggest that the strongest risk markers identified here may reflect patterns most characteristic of Western, industrialized settings. Future research should systematically include underrepresented groups, collect standardized diversity data, and examine how cultural norms, gender roles, and structural inequities shape dyadic and situational processes associated with IPV.

Structural conditions were not represented consistently enough in the eligible meta-analyses to support inclusion in the effect-size synthesis or tables. Nevertheless, broader evidence indicates that economic dependence may limit the material resources needed to seek safety, while food and housing insecurity have been associated with IPV victimization, although the temporal direction of these associations remains uncertain (Breiding et al., 2017). For immigrant women and women from racial or ethnic minority groups, precarious immigration status and immigration-related legal constraints, language barriers, racism, social isolation, and limited culturally and linguistically responsive services may impede help-seeking and leaving abusive relationships (Hulley et al., 2023). These factors should be understood as structural and institutional constraints rather than victim characteristics that cause IPV. Their limited representation in the eligible meta-analytic literature constitutes an important evidence gap and priority for future research.

### 7.3. Methodological Limitations

This review has several methodological limitations. Because the review was selective and conceptually focused, a reproducible database-by-database search log and initial retrieval and deduplication counts were not prospectively archived, limiting reproducibility. The included meta-analytic reviews differed in their sampling frames, operational definitions, measurement approaches, and effect-size metrics. They also varied in the methodological safeguards they reported. Some source reviews formally appraised primary-study quality and conducted sensitivity analyses (Oram et al., 2014; Stith et al., 2004). Several reviews also reported statistical assessments of heterogeneity and/or publication bias (Cafferky et al., 2018; Spencer et al., 2016, 2019a, 2019b, 2022). The present review did not independently apply a standardized appraisal of the methodological quality or risk of bias of the included meta-analytic reviews. The extent of primary-study overlap across the source reviews was also not quantified; consequently, the reviews should not be assumed to represent fully independent bodies of evidence. The summarized effect sizes should therefore be interpreted as recurring patterns of association within and across the source reviews rather than as fully independent or directly comparable estimates of equal certainty. Several source reviews explicitly characterized their findings as correlational or cautioned that temporal ordering could not be established (Cafferky et al., 2018; Oram et al., 2014, Spencer et al., 2019a, 2019b, 2022). Accordingly, pooled estimates were treated as associations unless a source review explicitly reported prospective, time-ordered evidence. Prospective association alone was not interpreted as evidence of causation or a mechanism of escalation. These limitations constrain the certainty and comparability of individual estimates but do not preclude the conceptual integration of recurrent findings across the reviewed evidence. A future systematic umbrella review should apply a standardized appraisal of review quality and risk of bias, quantify primary-study overlap, and incorporate heterogeneity and publication-bias findings when weighing the evidence.

### 7.4. Conclusions

Although the dyadic model highlights interpersonal processes within relationships, individuals experiencing IPV may face substantial systemic and structural barriers that constrain their ability to alter those dynamics or leave the relationship. Understanding risk markers across multiple levels may inform the development of more context-sensitive interventions.

Clinicians may use these levels as an organizational aid during assessment and treatment planning to distinguish background and dispositional vulnerabilities, ongoing relationship-context markers, and acute situational conditions. The framework should supplement rather than replace validated risk-assessment procedures. Depending on the pattern of violence and after careful assessment of coercive control and safety, interventions may address hostile attributional processes and difficulties in communication and conflict management. Couple-focused approaches should not be assumed appropriate in relationships characterized by coercive control or substantial safety risk.

Overall, interventions should be tailored to the pattern and context of violence, with victim safety and perpetrator accountability remaining central.

## Figures and Tables

**Table 1:** Characteristics of the eight included meta-analyses.

First Author, Year	Type of IPV	Focus of Study	Sample Characteristics	Number of Studies Included	Total Effect Sizes	Risk Markers Examined	Effect Size Measure	Gender Differences	Time Frame
Stith et al. (2004)	Physical abuse	Perpetration & Victimization	Heterosexual married and/or cohabiting couples	85	308 effect sizes	16 perpetration and 9 victimization risk markers	Correlation coefficient (*r*)	Gender differences reported only for the association between marital satisfaction and perpetration because data were insufficient for women who perpetrated IPV and men who experienced IPV	1980–2000
Stith et al. (2008)	Physical aggression/violence	Perpetration & Victimization	Heterosexual married and/or cohabiting couples	32	49 effect sizes	Marital satisfaction and marital discord	Correlation coefficient (*r*)	Gender differences reported for perpetration and victimization estimates	1981–2005
Oram et al. (2014)	Physical violence ^b^	Perpetration	Men and women aged ≥16 years with diagnosed psychiatric disorders	17	11 effect sizes for lifetime physical violence against a partner	Psychiatric disorders, including schizophrenia, bipolar disorder, depression, PTSD, GAD, panic disorder, dysthymia, and social phobia	Odds ratio (OR)	Higher estimates reported for men than for women	1994–2012
Spencer et al. (2016)	Physical IPV	Perpetration	Adult heterosexual married or cohabiting couples	580	520 effect sizes ^a^	60 different risk markers	Correlation coefficient (*r*)	Gender differences reported for 3 of 60 risk markers	1980–2012
Cafferky et al. (2018)	Physical aggression/violence	Perpetration & Victimization	Adults in heterosexual relationships	285	983 effect sizes	Substance use (overall), alcohol use, and drug use (various types)	Correlation coefficient (*r*)	Gender differences reported for 3 of 6 risk markers	1980–2013
Spencer et al. (2019a)	Physical IPV	Perpetration & Victimization	Ongoing adult romantic relationships	207	511 effect sizes	Depression, anxiety, PTSD, BPD, and APD	Correlation coefficient (*r*)	Gender differences reported for 1 of 5 risk markers	1980–2014
Spencer et al. (2019b)	Physical IPV	Victimization	Adult heterosexual men and women in married or cohabiting relationships	391	1731 effect sizes	50 risk markers for women, 28 risk markers for men	Correlation coefficient (*r*)	Gender differences reported for 5 of 28 risk markers	1980–2016
Spencer et al. (2022)	Physical IPV	Perpetration	Adults in opposite-sex relationships	503	2972 effect sizes	63 different risk markers	Correlation coefficient (*r*)	Gender differences reported for 9 of 44 risk markers	1980–2018

*Note.* BPD = borderline personality disorder; APD = antisocial personality disorder; PTSD = posttraumatic stress disorder; GAD = generalized anxiety disorder. ^a^ The number of effect sizes includes only those for the three risk markers that significantly differed between men and women. ^b^ Although this study examined various forms of IPV, the results focus on physical IPV due to a lack of data for other IPV types.

**Table 2:** Background and dispositional risk markers for physical IPV.

Risk Marker	Effect Size	IPV Role	Gender Group	Source
Generalized anxiety disorder	0.53	Perpetration	Men ^a^	Oram et al. (2014) ^c^
Depression	0.49	Perpetration	Men	Oram et al. (2014) ^c^
History of abuse in past relationships	0.49	Victimization	Men	Spencer et al. (2019b)
Panic disorder	0.45	Perpetration	Men	Oram et al. (2014) ^c^
Depression	0.43	Perpetration	Women	Oram et al. (2014) ^c^
Generalized anxiety disorder	0.43	Perpetration	Women	Oram et al. (2014) ^c^
Borderline personality disorder	0.34	Perpetration	Combined ^b^	Spencer et al. (2019a)
Posttraumatic stress disorder	0.34	Victimization	Combined	Spencer et al. (2019a)
Posttraumatic stress disorder	0.34	Victimization	Women	Spencer et al. (2019b)
Panic disorder	0.33	Perpetration	Women	Oram et al. (2014) ^c^
Anger	0.32	Perpetration	Combined	Spencer et al. (2022)
Illicit drug use	0.31	Perpetration	Men	Stith et al. (2004)
Traditional sex-role ideology	0.29	Perpetration	Men	Stith et al. (2004)
Depression	0.29	Victimization	Women	Spencer et al. (2019b)
Posttraumatic stress disorder	0.29	Victimization	Men	Spencer et al. (2019b)
Fear	0.29	Victimization	Women	Spencer et al. (2019b)
Depression	0.28	Victimization	Women	Stith et al. (2004)
Antisocial personality disorder	0.27	Perpetration	Combined	Spencer et al. (2019a)
Antisocial personality disorder	0.27	Perpetration	Combined	Spencer et al. (2022)
General mental health problems	0.27	Perpetration	Combined	Spencer et al. (2022)
Fear of partner violence	0.27	Victimization	Women	Stith et al. (2004)
Borderline personality disorder	0.27	Victimization	Men	Spencer et al. (2019b)
History of abuse in past relationships	0.27	Victimization	Women	Spencer et al. (2019b)
Anger/hostility	0.26	Perpetration	Men	Stith et al. (2004)
Narcissism	0.26	Perpetration	Combined	Spencer et al. (2022)
External locus of control	0.26	Perpetration	Combined	Spencer et al. (2022)
Prior arrest	0.26	Perpetration	Combined	Spencer et al. (2022)
Drug use	0.25	Perpetration	Combined	Spencer et al. (2022)
Witnessing parental IPV	0.25	Perpetration	Men	Spencer et al. (2022)
Depression	0.25	Victimization	Combined	Spencer et al. (2019a)
Drug use	0.25	Victimization	Women	Spencer et al. (2019b)
Witnessing IPV and experiencing child physical abuse	0.25	Perpetration	Men	Spencer et al. (2016)
Alcohol abuse	0.24	Perpetration	Men	Stith et al. (2004)
Access to weapons	0.24	Perpetration	Combined	Spencer et al. (2022)
History of being abused as a child	0.24	Perpetration	Men	Spencer et al. (2022)
Stress	0.24	Victimization	Men	Spencer et al. (2019b)
Depression	0.23	Perpetration	Men	Stith et al. (2004)
Substance use (alcohol and drug use)	0.23	Perpetration	Men	Spencer et al. (2022)
Antisocial personality disorder	0.23	Victimization	Women	Spencer et al. (2019b)
Childhood abuse in the family of origin	0.23	Victimization	Women	Spencer et al. (2019b)
Substance use (alcohol + drug)	0.23	Perpetration	Men	Cafferky et al. (2018)
Substance use (alcohol and drug use)	0.22	Perpetration	Combined	Spencer et al. (2022)
History of being abused as a child	0.22	Perpetration	Combined	Spencer et al. (2022)
Witnessing parental IPV	0.22	Perpetration	Combined	Spencer et al. (2022)
Depression	0.22	Perpetration	Combined	Spencer et al. (2022)
Alcohol use	0.22	Perpetration	Men	Spencer et al. (2022)
Anger	0.22	Victimization	Men	Spencer et al. (2019b)
Antisocial personality disorder	0.22	Victimization	Men	Spencer et al. (2019b)
Substance use	0.22	Victimization	Women	Spencer et al. (2019b)
Alcohol use	0.22	Perpetration	Men	Cafferky et al. (2018)
Alcohol use	0.22	Perpetration	Men	Spencer et al. (2016)
Posttraumatic stress disorder	0.21	Perpetration	Combined	Spencer et al. (2019a)
Depression	0.21	Perpetration	Combined	Spencer et al. (2019a)
Alcohol use	0.21	Perpetration	Combined	Spencer et al. (2022)
Posttraumatic stress disorder	0.21	Perpetration	Combined	Spencer et al. (2022)
Impulsivity	0.21	Perpetration	Combined	Spencer et al. (2022)
Drug use	0.21	Victimization	Men	Spencer et al. (2019b)
Alcohol use	0.21	Victimization	Women	Spencer et al. (2019b)
Anger	0.21	Victimization	Women	Spencer et al. (2019b)
Substance use (alcohol + drug)	0.21	Victimization	Women	Cafferky et al. (2018)
Adherence to traditional gender roles	0.20	Perpetration	Combined	Spencer et al. (2022)
Witnessing IPV in the family of origin	0.20	Victimization	Women	Spencer et al. (2019b)
Borderline personality disorder	0.20	Victimization	Women	Spencer et al. (2019b)
Anxious attachment style	0.20	Victimization	Women	Spencer et al. (2019b)
Anxiety	0.19	Victimization	Combined	Spencer et al. (2019a)
Borderline personality disorder	0.19	Victimization	Combined	Spencer et al. (2019a)
Witnessing IPV and experiencing child physical abuse	0.19	Perpetration	Women	Spencer et al. (2016)
Trauma	0.18	Perpetration	Combined	Spencer et al. (2022)
Antisocial personality disorder	0.18	Victimization	Combined	Spencer et al. (2019a)
Depression	0.18	Victimization	Men	Spencer et al. (2019b)
Substance use (alcohol and drug use)	0.17	Perpetration	Women	Spencer et al. (2022)
Witnessing parental IPV	0.17	Perpetration	Women	Spencer et al. (2022)
Witnessing IPV in the family of origin	0.17	Victimization	Men	Spencer et al. (2019b)
Approval of violence	0.17	Victimization	Women	Spencer et al. (2019b)
Substance use (alcohol + drug)	0.17	Perpetration	Women	Cafferky et al. (2018)
Substance use (alcohol + drug)	0.17	Victimization	Men	Cafferky et al. (2018)
Alcohol use	0.16	Perpetration	Women	Spencer et al. (2022)
Anxiety	0.16	Perpetration	Combined	Spencer et al. (2022)
Stress	0.16	Perpetration	Combined	Spencer et al. (2022)
Anxious attachment	0.16	Perpetration	Combined	Spencer et al. (2022)
Anxiety	0.16	Victimization	Men	Spencer et al. (2019b)
Stress	0.16	Victimization	Women	Spencer et al. (2019b)
Experiencing childhood abuse within the family	0.15	Victimization	Men	Spencer et al. (2019b)
Alcohol use	0.15	Perpetration	Women	Cafferky et al. (2018)
Alcohol use	0.15	Perpetration	Women	Spencer et al. (2016)
History of being abused as a child	0.14	Perpetration	Women	Spencer et al. (2022)
Alcohol use	0.14	Victimization	Men	Spencer et al. (2019b)
Mental health problems	0.14	Victimization	Women	Spencer et al. (2019b)
Anxiety	0.13	Perpetration	Combined	Spencer et al. (2019a)
Avoidant attachment	0.13	Perpetration	Combined	Spencer et al. (2022)
Alcohol abuse	0.13	Victimization	Women	Stith et al. (2004)
Impulsivity	0.13	Victimization	Men	Spencer et al. (2019b)
Disorganized attachment	0.11	Perpetration	Combined	Spencer et al. (2022)
Physical health problems	0.11	Perpetration	Combined	Spencer et al. (2022)
Traditional gender roles	0.08	Victimization	Women	Spencer et al. (2019b)
Impulsivity	0.07	Victimization	Women	Spencer et al. (2019b)
Internal locus of control	−0.30	Perpetration	Men	Spencer et al. (2022)
Higher income	−0.17	Perpetration	Combined	Spencer et al. (2022)
Higher education	−0.15	Perpetration	Men	Spencer et al. (2022)
Higher education	−0.14	Perpetration	Combined	Spencer et al. (2022)
Higher levels of self-esteem	−0.14	Perpetration	Combined	Spencer et al. (2022)
Older age	−0.13	Victimization	Men	Spencer et al. (2019b)
Secure attachment	−0.11	Perpetration	Combined	Spencer et al. (2022)
Older age	−0.10	Perpetration	Combined	Spencer et al. (2022)
Religiosity	−0.09	Perpetration	Men	Spencer et al. (2022)
Higher levels of education	−0.09	Victimization	Women	Spencer et al. (2019b)
Employment	−0.07	Perpetration	Combined	Spencer et al. (2022)
Religiosity	−0.07	Perpetration	Combined	Spencer et al. (2022)
Older age	−0.02	Victimization	Women	Spencer et al. (2019b)
General mental health problems	Nonsignificant	Perpetration	Women	Spencer et al. (2022)
Approval of violence	Nonsignificant	Perpetration	Women	Spencer et al. (2022)
Higher levels of self-esteem	Nonsignificant	Perpetration	Women	Spencer et al. (2022)
Employment	Nonsignificant	Perpetration	Women	Spencer et al. (2022)
Religiosity	Nonsignificant	Perpetration	Women	Spencer et al. (2022)
Level of education	Nonsignificant	Victimization	Men	Spencer et al. (2019b)
Employment status	Nonsignificant	Victimization	Men	Spencer et al. (2019b)
Mental health problems	Nonsignificant	Victimization	Men	Spencer et al. (2019b)
Secure attachment	Nonsignificant	Victimization	Women	Spencer et al. (2019b)
Avoidant attachment	Nonsignificant	Victimization	Women	Spencer et al. (2019b)
Physical health	Nonsignificant	Victimization	Women	Spencer et al. (2019b)
Self-blame	Nonsignificant	Victimization	Women	Spencer et al. (2019b)
Self-esteem	Nonsignificant	Victimization	Women	Spencer et al. (2019b)
Trauma	Nonsignificant	Victimization	Women	Spencer et al. (2019b)
Prior arrest	Nonsignificant	Victimization	Women	Spencer et al. (2019b)
Religiosity	Nonsignificant	Victimization	Women	Spencer et al. (2019b)
Employment	Nonsignificant	Victimization	Women	Spencer et al. (2019b)
Drug use	Not reported	Victimization	Men/Women	Cafferky et al. (2018)
Age	Not reported	Perpetration	Men/Women	Spencer et al. (2016)
Education	Not reported	Perpetration	Men/Women	Spencer et al. (2016)
Income	Not reported	Perpetration	Men/Women	Spencer et al. (2016)
Employment status	Not reported	Perpetration	Men/Women	Spencer et al. (2016)
Depression	Not reported	Perpetration	Men/Women	Spencer et al. (2016)
Anxiety	Not reported	Perpetration	Men/Women	Spencer et al. (2016)
Anger	Not reported	Perpetration	Men/Women	Spencer et al. (2016)
Stress	Not reported	Perpetration	Men/Women	Spencer et al. (2016)
Trauma	Not reported	Perpetration	Men/Women	Spencer et al. (2016)
Self-esteem	Not reported	Perpetration	Men/Women	Spencer et al. (2016)
Posttraumatic stress disorder	Not reported	Perpetration	Men/Women	Spencer et al. (2016)
Antisocial personality disorder	Not reported	Perpetration	Men/Women	Spencer et al. (2016)
Borderline personality disorder	Not reported	Perpetration	Men/Women	Spencer et al. (2016)
General mental health	Not reported	Perpetration	Men/Women	Spencer et al. (2016)
Drug use	Not reported	Perpetration	Men/Women	Spencer et al. (2016)
Alcohol difficulties	Not reported	Perpetration	Men/Women	Spencer et al. (2016)
Physical health status	Not reported	Perpetration	Men/Women	Spencer et al. (2016)
History of spousal abuse	Not reported	Perpetration	Men/Women	Spencer et al. (2016)
Internal locus of control	Not reported	Perpetration	Men/Women	Spencer et al. (2016)
Approval of violence	Not reported	Perpetration	Men/Women	Spencer et al. (2016)
Religiosity	Not reported	Perpetration	Men/Women	Spencer et al. (2016)
Coping skills	Not reported	Perpetration	Men/Women	Spencer et al. (2016)
Impulsivity	Not reported	Perpetration	Men/Women	Spencer et al. (2016)
Belief in male privilege	Not reported	Perpetration	Men/Women	Spencer et al. (2016)
Prior arrest	Not reported	Perpetration	Men/Women	Spencer et al. (2016)
Attachment styles	Not reported	Perpetration	Men/Women	Spencer et al. (2016)
Self-blame	Not reported	Perpetration	Men/Women	Spencer et al. (2016)
Blaming others for IPV	Not reported	Perpetration	Men/Women	Spencer et al. (2016)
Witnessing parental domestic violence	Not reported	Perpetration	Men/Women	Spencer et al. (2016)
Observing mother hitting father	Not reported	Perpetration	Men/Women	Spencer et al. (2016)
Observing father hitting mother	Not reported	Perpetration	Men/Women	Spencer et al. (2016)
Experiencing physical abuse during childhood	Not reported	Perpetration	Men/Women	Spencer et al. (2016)
Paternal child abuse	Not reported	Perpetration	Men/Women	Spencer et al. (2016)
Maternal child abuse	Not reported	Perpetration	Men/Women	Spencer et al. (2016)

*Note.* ^a^ Effect sizes were considered separately for men and women. ^b^ Effect sizes were aggregated for men and women. ^c^ In this review, effect sizes originally reported as odds ratios were transformed into correlation coefficients for consistency and comparability. However, the results are approximate and should be interpreted with caution. Entries shown as “Men/Women” indicate that the marker was examined separately for men and women, but the corresponding effect sizes were not reported.

**Table 3:** Relationship-context risk markers for physical IPV.

Risk Marker	Effect Size	IPV Role	Gender Group	Source
Previous injury	0.64	Victimization	Men ^a^	Spencer et al. (2019b)
Caused previous injuries to partner	0.58	Perpetration	Combined ^b^	Spencer et al. (2022)
Previous physical IPV perpetration	0.56	Victimization	Women	Spencer et al. (2019b)
Previous injury	0.54	Victimization	Women	Spencer et al. (2019b)
Emotional IPV perpetration	0.53	Perpetration	Combined	Spencer et al. (2022)
Emotional abuse victimization	0.53	Victimization	Men	Spencer et al. (2019b)
Physical IPV victimization	0.52	Perpetration	Combined	Spencer et al. (2022)
Emotional abuse victimization	0.51	Victimization	Women	Spencer et al. (2019b)
Emotional/verbal abuse	0.49	Perpetration	Men	Stith et al. (2004)
Previous physical IPV perpetration	0.49	Victimization	Men	Spencer et al. (2019b)
Stalking perpetration	0.47	Perpetration	Men	Spencer et al. (2022)
Emotional IPV victimization	0.44	Perpetration	Combined	Spencer et al. (2022)
Sexual abuse victimization	0.44	Perpetration	Combined	Spencer et al. (2022)
Forced-sex/sexual IPV victimization	0.44	Victimization	Women	Spencer et al. (2019b)
Emotional abuse perpetration	0.42	Victimization	Men	Spencer et al. (2019b)
Previous physical IPV perpetration	0.42	Perpetration	Combined	Spencer et al. (2022)
Demand/withdraw communication patterns	0.42	Perpetration	Men	Spencer et al. (2022)
Demand/withdraw communication patterns	0.41	Perpetration	Men	Spencer et al. (2016)
Emotional abuse perpetration	0.41	Victimization	Women	Spencer et al. (2019b)
Stalking victimization ^c^	0.40	Victimization	Women	Spencer et al. (2019b)
Threatens to harm self ^c^	0.39	Victimization	Women	Spencer et al. (2019b)
Demand/withdraw communication patterns	0.37	Perpetration	Combined	Spencer et al. (2022)
Demand/withdraw ^c^	0.32	Victimization	Women	Spencer et al. (2019b)
Perpetrator’s controlling behaviors ^c^	0.31	Victimization	Women	Spencer et al. (2019b)
Controlling behaviors	0.30	Perpetration	Combined	Spencer et al. (2022)
Relationship dissatisfaction	0.27	Victimization	Women	Spencer et al. (2019b)
Perpetrator’s power in the relationship	0.25	Victimization	Women	Spencer et al. (2019b)
History of partner abuse	0.24	Perpetration	Men	Stith et al. (2004)
Relationship dissatisfaction	0.23	Victimization	Men	Spencer et al. (2019b)
Power in relationship	0.18	Perpetration	Combined	Spencer et al. (2022)
Demand/withdraw communication patterns	0.16	Perpetration	Women	Spencer et al. (2022)
Demand/withdraw communication patterns	0.16	Perpetration	Women	Spencer et al. (2016)
Marital satisfaction	−0.41	Victimization	Women	Stith et al. (2008)
Marital satisfaction	−0.40	Victimization	Combined	Stith et al. (2008)
Marital satisfaction	−0.30	Perpetration	Men	Stith et al. (2004)
Marital satisfaction	−0.30	Victimization	Men	Stith et al. (2008)
Marital satisfaction	−0.28	Perpetration	Men	Stith et al. (2008)
Marital satisfaction	−0.27	Perpetration	Combined	Stith et al. (2008)
Marital discord	−0.27	Perpetration	Combined	Stith et al. (2008)
Marital satisfaction	−0.27	Perpetration	Combined	Stith et al. (2008)
Marital satisfaction	−0.25	Perpetration	Women	Stith et al. (2004)
Relationship satisfaction	−0.25	Perpetration	Combined	Spencer et al. (2022)
Communication skills	−0.24	Perpetration	Combined	Spencer et al. (2022)
Marital satisfaction	−0.21	Perpetration	Women	Stith et al. (2008)
Coping skills	−0.20	Perpetration	Combined	Spencer et al. (2022)
Conflict resolution skills	−0.17	Perpetration	Combined	Spencer et al. (2022)
Relationship communication skills	−0.17	Victimization	Women	Spencer et al. (2019b)
Length of time living together	−0.16	Perpetration	Combined	Spencer et al. (2022)
Empathy	−0.14	Perpetration	Men	Spencer et al. (2022)
Length of relationship	−0.11	Perpetration	Combined	Spencer et al. (2022)
Marital status	Nonsignificant	Perpetration	Combined	Spencer et al. (2022)
Victim of forced-sex/sexual IPV victimization	Nonsignificant	Victimization	Men	Spencer et al. (2019b)
Perpetrator’s power in the relationship	Nonsignificant	Victimization	Men	Spencer et al. (2019b)
Marital status	Nonsignificant	Victimization	Men	Spencer et al. (2019b)
Length of relationship	Nonsignificant	Victimization	Men	Spencer et al. (2019b)
Conflict resolution skills	Nonsignificant	Victimization	Women	Spencer et al. (2019b)
Length of relationship	Nonsignificant	Victimization	Women	Spencer et al. (2019b)
Marital status (married or divorced)	Not reported	Perpetration	Men/Women	Spencer et al. (2016)
Relationship length	Not reported	Perpetration	Men/Women	Spencer et al. (2016)
Relational satisfaction	Not reported	Perpetration	Men/Women	Spencer et al. (2016)
Relational distress	Not reported	Perpetration	Men/Women	Spencer et al. (2016)
Communication patterns	Not reported	Perpetration	Men/Women	Spencer et al. (2016)
Conflict resolution skills	Not reported	Perpetration	Men/Women	Spencer et al. (2016)
Psychological/emotional abuse	Not reported	Perpetration	Men/Women	Spencer et al. (2016)
Approval of violence in the relationship	Not reported	Perpetration	Men/Women	Spencer et al. (2016)
Previous violence toward this partner	Not reported	Perpetration	Men/Women	Spencer et al. (2016)
Previous victimization by this partner	Not reported	Perpetration	Men/Women	Spencer et al. (2016)
Caused injury	Not reported	Perpetration	Men/Women	Spencer et al. (2016)
Weapon use in previous violent incidents with the partner	Not reported	Perpetration	Men/Women	Spencer et al. (2016)
Property destruction in previous violent incidents with the partner	Not reported	Perpetration	Men/Women	Spencer et al. (2016)

*Note.* ^a^ Effect sizes were considered separately for men and women. ^b^ Effect sizes were aggregated for men and women. ^c^ Risk marker reported only for women’s victimization, not men’s victimization. Entries shown as “Men/Women” indicate that the marker was examined separately for men and women, but the corresponding effect sizes were not reported.

**Table 4:** Situational-context risk markers for physical IPV.

Risk Marker	Effect Size	IPV Role	Gender Group	Source
Threatens to harm partner	0.49	Perpetration	Combined ^a^	Spencer et al. (2022)
Forced sex	0.45	Perpetration	Men ^b^	Stith et al. (2004)
Verbal arguments	0.43	Perpetration	Combined	Spencer et al. (2022)
IPV perpetration against partner	0.41	Victimization	Women	Stith et al. (2004)
Threatens to harm self	0.39	Victimization	Women	Spencer et al. (2019b)
Violence toward non-family members	0.28	Perpetration	Combined	Spencer et al. (2022)
Career/life stress	0.26	Perpetration	Men	Stith et al. (2004)
Jealousy	0.24	Perpetration	Combined	Spencer et al. (2022)
Perpetrator’s jealousy	0.23	Victimization	Women	Spencer et al. (2019b)
Infidelity	0.22	Perpetration	Men	Spencer et al. (2022)
Separation	0.21	Victimization	Women	Spencer et al. (2019b)
Jealousy	0.17	Perpetration	Men	Stith et al. (2004)
Physical abuse of own children	0.17	Perpetration	Combined	Spencer et al. (2022)
Being accused of infidelity	0.14	Victimization	Women	Spencer et al. (2019b)
Financial stress	0.11	Perpetration	Combined	Spencer et al. (2022)
Threatens to harm self	0.10	Perpetration	Men	Spencer et al. (2022)
Number of children	0.08	Perpetration	Combined	Spencer et al. (2022)
Number of children	0.07	Victimization	Women	Spencer et al. (2019b)
Social support	Not reported	Perpetration	Men/Women	Spencer et al. (2016)
Physical abuse of own children	Not reported	Perpetration	Men/Women	Spencer et al. (2016)
Financial stress	Not reported	Perpetration	Men/Women	Spencer et al. (2016)
Violence toward non-family members	Not reported	Perpetration	Men/Women	Spencer et al. (2016)
Number of children	Not reported	Perpetration	Men/Women	Spencer et al. (2016)
Verbal arguments	Not reported	Perpetration	Men/Women	Spencer et al. (2016)
Forced sex	Not reported	Perpetration	Men/Women	Spencer et al. (2016)
Number of children	Not reported	Perpetration	Men/Women	Spencer et al. (2016)
Separation	Not reported	Perpetration	Men/Women	Spencer et al. (2016)

*Note.* ^a^ Effect sizes labeled “Combined” were aggregated across men and women. ^b^ Where separate estimates were reported, the gender group is identified as “Men” or “Women.” Entries shown as “Men/Women” indicate that the marker was examined separately for men and women, but the corresponding effect sizes were not reported.

## Data Availability

No new data were created or analyzed in this study. Data sharing is not applicable to this article.

## Abbreviations

The following abbreviations are used in this manuscript:

IPV	Intimate Partner Violence
CDC	Centers for Disease Control and Prevention
WHO	World Health Organization
BPD	Borderline Personality Disorder
APD	Antisocial Personality Disorder
PTSD	Posttraumatic Stress Disorder
GAD	Generalized Anxiety Disorder
OR	Odds Ratio

## References

[B1-behavsci-16-01468] Andersen S. M., Miranda R., Krueger R. F., Tackett J. L. (2006). Through the lens of the relational self: Triggering emotional suffering in the social-cognitive process of transference. Personality and psychopathology.

[B2-behavsci-16-01468] Bartholomew K., Cobb R. J., Horowitz L. M., Strack S. (2011). Conceptualizing relationship violence as a dyadic process. Handbook of interpersonal psychology: Theory, research, assessment, and therapeutic interventions.

[B3-behavsci-16-01468] Behrman J., Frye M. (2021). Attitudes toward intimate partner violence in dyadic perspective: Evidence from Sub-Saharan Africa. Demography.

[B4-behavsci-16-01468] Breiding M. J., Basile K. C., Klevens J., Smith S. G. (2017). Economic insecurity and intimate partner and sexual violence victimization. American Journal of Preventive Medicine.

[B5-behavsci-16-01468] Breiding M. J., Basile K. C., Smith S. G., Black M. C., Mahendra R. R. (2015). Intimate partner violence surveillance: Uniform definitions and recommended data elements, version 2.0 *(Tech. Rep.)*.

[B6-behavsci-16-01468] Cafferky B. M., Mendez M., Anderson J. R., Stith S. M. (2018). Substance use and intimate partner violence: A meta-analytic review. Psychology of Violence.

[B7-behavsci-16-01468] Caldwell J. E., Swan S. C., Woodbrown V. D. (2012). Gender differences in intimate partner violence outcomes. Psychology of Violence.

[B8-behavsci-16-01468] Carson R. C., Anchin J. C., Kiesler D. J. (1982). Self-fulfilling prophecy, maladaptive behavior, and psychotherapy. Handbook of interpersonal psychotherapy.

[B9-behavsci-16-01468] Conroy A. A. (2014). Gender, power, and intimate partner violence: A study on couples from rural Malawi. Journal of Interpersonal Violence.

[B10-behavsci-16-01468] Costa B. M., Kaestle C. E., Walker A., Curtis A., Day A., Toumbourou J. W., Miller P. (2015). Longitudinal predictors of domestic violence perpetration and victimization: A systematic review. Aggression and Violent Behavior.

[B11-behavsci-16-01468] Curtis A., Harries T., Pizzirani B., Hyder S., Baldwin R., Mayshak R., Walker A., Toumbourou J. W., Miller P. (2023). Childhood predictors of adult intimate partner violence perpetration and victimization: A systematic review. Journal of Family Violence.

[B12-behavsci-16-01468] Devries K. M., Mak J. Y., Bacchus L. J., Child J. C., Falder G., Petzold M., Astbury J., Watts C. H. (2013). Intimate partner violence and incident depressive symptoms and suicide attempts: A systematic review of longitudinal studies. PLoS Medicine.

[B13-behavsci-16-01468] Dutton D. G. (1995). The domestic assault of women: Psychological and criminal justice perspectives.

[B14-behavsci-16-01468] Ghuman S. J., Lee H. J., Smith H. L. (2006). Measurement of women’s autonomy according to women and their husbands: Results from five Asian countries. Social Science Research.

[B15-behavsci-16-01468] Horowitz L. M., Strack S. (2011). Handbook of interpersonal psychology: Theory, research, assessment, and therapeutic interventions.

[B16-behavsci-16-01468] Hulley J., Bailey L., Kirkman G., Gibbs G. R., Gomersall T., Latif A., Jones A. (2023). Intimate partner violence and barriers to help-seeking among Black, Aasian, minority ethnic and immigrant women: A qualitative metasynthesis of global research. Trauma, Violence, & Abuse.

[B17-behavsci-16-01468] Johnson M. P. (2008). A typology of domestic violence: Intimate terrorism, violent resistance, and situational couple violence.

[B18-behavsci-16-01468] Jonas S., Khalifeh H., Bebbington P. E., McManus S., Brugha T., Meltzer H., Howard L. M. (2014). Gender differences in intimate partner violence and psychiatric disorders in England: Results from the 2007 adult psychiatric morbidity survey. Epidemiology and Psychiatric Sciences.

[B19-behavsci-16-01468] Khalifeh H., Dean K. (2010). Gender and violence against people with severe mental illness. International Review of Psychiatry.

[B20-behavsci-16-01468] Kiesler D. J. (1983). The 1982 interpersonal circle: A taxonomy for complementarity in human transactions. Psychological Review.

[B21-behavsci-16-01468] Kraemer H. C., Kazdin A. E., Offord D. R., Kessler R. C., Jensen P. S., Kupfer D. J. (1997). Coming to terms with the terms of risk. Archives of General Psychiatry.

[B22-behavsci-16-01468] Machado A., Sousa C., Cunha O. (2024). Bidirectional violence in intimate relationships: A systematic review. Trauma, Violence & Abuse.

[B23-behavsci-16-01468] Oram S., Trevillion K., Khalifeh H., Feder G., Howard L. M. (2014). Systematic review and meta-analysis of psychiatric disorder and the perpetration of partner violence. Epidemiology and Psychiatric Sciences.

[B24-behavsci-16-01468] Rodriguez L. M., Straus M. A., Sturmey P., Sturmey P. (2017). Dyadic conceptualization, measurement, and analysis of family violence. The wiley handbook of violence and aggression.

[B25-behavsci-16-01468] Spencer C. M., Cafferky B., Stith S. M. (2016). Gender differences in risk markers for perpetration of physical partner violence: Results from a meta-analytic review. Journal of Family Violence.

[B26-behavsci-16-01468] Spencer C. M., Mallory A. B., Cafferky B. M., Kimmes J. G., Beck A. R., Stith S. M. (2019a). Mental health factors and intimate partner violence perpetration and victimization: A meta-analysis. Psychology of Violence.

[B27-behavsci-16-01468] Spencer C. M., Stith S. M., Cafferky B. (2019b). Risk markers for physical intimate partner violence victimization: A meta-analysis. Aggression and Violent Behavior.

[B28-behavsci-16-01468] Spencer C. M., Stith S. M., Cafferky B. (2022). What puts individuals at risk for physical intimate partner violence perpetration? A meta-analysis examining risk markers for men and women. Trauma, Violence, & Abuse.

[B29-behavsci-16-01468] Stith S. M., Green N. M., Smith D. B., Ward D. B. (2008). Marital satisfaction and discord as risk markers for intimate partner violence: A meta-analytical review. Journal of Family Violence.

[B30-behavsci-16-01468] Stith S. M., Rosen K. H., Middleton K. A., Busch A. L., Lundeberg K., Carlton R. P. (2000). The intergenerational transmission of spouse abuse: A meta-analysis. Journal of Marriage and Family.

[B31-behavsci-16-01468] Stith S. M., Smith D. B., Penn C. E., Ward D. B., Tritt D. (2004). Intimate partner physical abuse perpetration and victimization risk factors: A meta-analytic review. Aggression and Violent Behavior.

[B32-behavsci-16-01468] Sugarman D. B., Frankel S. L. (1996). Patriarchal ideology and wife-assault: A meta-analytic review. Journal of Family Violence.

[B33-behavsci-16-01468] Teplin L. A., McClelland G. M., Abram K. M., Weiner D. A. (2005). Crime victimization in adults with severe mental illness: Comparison with the national crime victimization survey. Archives of General Psychiatry.

[B34-behavsci-16-01468] Tolin D. F., Foa E. B. (2006). Sex differences in trauma and posttraumatic stress disorder: A quantitative review of 25 years of research. Psychological Bulletin.

[B35-behavsci-16-01468] Trevillion K., Oram S., Feder G., Howard L. M. (2012). Experiences of domestic violence and mental disorders: A systematic review and meta-analysis. PLoS ONE.

[B36-behavsci-16-01468] World Health Organization (2012). Understanding and addressing violence against women: Intimate partner violence *(Tech. Rep. No. WHO/RHR/12.36)*.

